# Electrochemical N–N Oxidatively Coupled Dehydrogenation of 3,5-Diamino-1*H*-1,2,4-triazole for Value-Added Chemicals and Bipolar Hydrogen Production

**DOI:** 10.1021/jacs.4c17225

**Published:** 2025-03-08

**Authors:** Jiachen Li, Yang Li, Yuqiang Ma, Zihang Zhao, Huarong Peng, Tao Zhou, Ming Xu, Daidi Fan, Haixia Ma, Jieshan Qiu, Zhengxiao Guo

**Affiliations:** † Xi’an Key Laboratory of Special Energy Materials, School of Chemical Engineering, Northwest University, Xi’an 710127, China; ‡ Department of Chemistry, The University of Hong Kong, Hong Kong 999077, SAR ,China; § State Key Laboratory of Chemical Resource Engineering, College of Chemical Engineering, Beijing University of Chemical Technology, Beijing 100029, China; ∥ Shaanxi Key Laboratory of Degradable Biomedical Materials, School of Chemical Engineering, Northwest University, Xi’an 710127, China; ⊥ College of Chemistry, Chemical Engineering and Resource Utilization, Center for Innovative Research in Synthetic Chemistry and Resource Utilization, Northeast Forestry University, Harbin 150040, China; # Zhijian Laboratory, Xi’an 710025, China

## Abstract

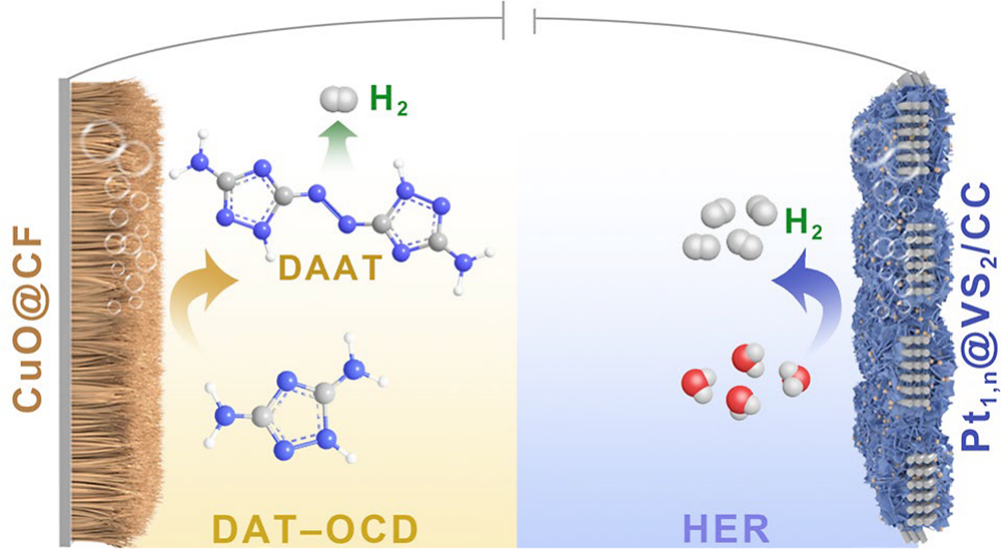

Electrochemical H_2_ production from water favors low-voltage molecular oxidation to replace the oxygen evolution reaction as an energy-saving and value-added approach. However, there exists a mismatch between the high demand for H_2_ and slow anodic reactions, restricting practical applications of such hybrid systems. Here, we propose a bipolar H_2_ production approach, with anodic H_2_ generation from the N–N oxidatively coupled dehydrogenation (OCD) of 3,5-diamino-1*H*-1,2,4-triazole (DAT), in addition to the cathodic H_2_ generation. The system requires relatively low oxidation potentials of 0.872 and 1.108 V vs RHE to reach 10 and 500 mA cm^–2^, respectively. The bipolar H_2_ production in an H-type electrolyzer requires only 0.946 and 1.129 V to deliver 10 and 100 mA cm^–2^, respectively, with the electricity consumption (1.3 kWh per m^3^ H_2_) reduced by 68%, compared with conventional water splitting. Moreover, the process is highly appealing due to the absence of traditional hazardous synthetic conditions of azo compounds at the anode and crossover/mixing of H_2_/O_2_ in the electrolyzer. A flow-type electrolyzer operates stably at 500 mA cm^–2^ for 300 h. Mechanistic studies reveal that the Pt single atom and nanoparticle (Pt_1,n_) optimize the adsorption of the S active sites for H_2_ production over the Pt_1,n_@VS_2_ cathodic catalysts. At the anode, the stepwise dehydrogenation of −NH_2_ in DAT and then oxidative coupling of −N–N– predominantly form azo compounds while generating H_2_. The present report paves a new way for atom-economical bipolar H_2_ production from N–N oxidative coupling of aminotriazole and green electrosynthesis of value-added azo chemicals.

## Introduction

Water electrolysis is an environmentally friendly pathway for hydrogen production, but direct coupling of the oxygen evolution reaction (OER) with the hydrogen evolution reaction (HER) results in several drawbacks. First, the anodic OER is thermodynamically unfavorable and sluggish due to the four-electron kinetic steps, responsible for 90% of the overall electricity consumption in water electrolysis.^1−3^ The practical input cell voltage exceeds 1.8 V with a high electricity consumption of ∼5 kWh/m^3^ of H_2_.^4,5^ Second, there is a high level of risk of H_2_ and O_2_ mixing, and thus, an expensive ion-exchange membrane is required to prevent gas crossover.^6^ Third, the anodic product, O_2_, is of low economic value. Finally, the anodic OER can also cause membrane degradation during the formation of reactive oxygen species.^7^ Hence, the alternative strategies of coupling thermodynamically more favorable oxidation reactions to replace the OER have been widely investigated in recent years. The substrate molecules include alcohols,^8−10^ aldehydes,^4,11,12^ and amines^13−15^ for upgrading to value-added chemicals. Other substrate molecules, such as urea,^16−18^ hydrazine,^19−22^ and sulfion,^23,24^ are considered for green electrochemical degradation of pollutant substances. Despite the significant advances in the coupled water electrolysis systems, these strategies are still in the infant stages of development and there are still important tasks to undertake: (1) to design cell configurations to meet the industrial-scale current densities (reach up to 500 mA cm^–2^); (2) to explore new green electrochemical pathways for pharmaceutical, energetic, and special chemicals;^7,25^ and (3) to produce H_2_ at both the cathode and the anode, so as to resolve the market-size mismatch between cathodic hydrogen production and anodic organic oxidation reaction.^4,11,12,26^

Azo compounds are ubiquitous for wide applications such as organic dyes, pigments, therapeutic reagents, and energetic materials.^27^ Amino triazoles are regarded as good energy-containing compounds with excellent thermal performance, stability, and high density.^28−30^ However, the traditional route of synthesizing azo compounds usually involves a high processing temperature and toxic/heavy metal reactants, accompanied by numerous steps; a viable alternative is the use of an electrochemical strategy.^31,32^ Organic electrochemistry can avoid the use of hazardous reagents and complex synthetic procedures, and it consumes only electrical energy, which increasingly comes from low-cost renewable sources.^33^ Hence, this work proposes a one-step electrochemical N–N oxidative-coupling dehydrogenation (OCD) of 3,5-diamino-1*H*-1,2,4-triazole (DAT-OCD) to synthesize 5,5′-diamino-3,3′-azo-1*H*-1,2,4-triazole (DAAT) compounds, while generating hydrogen from both electrodes, as outlined in the following.

**Scheme 1:**
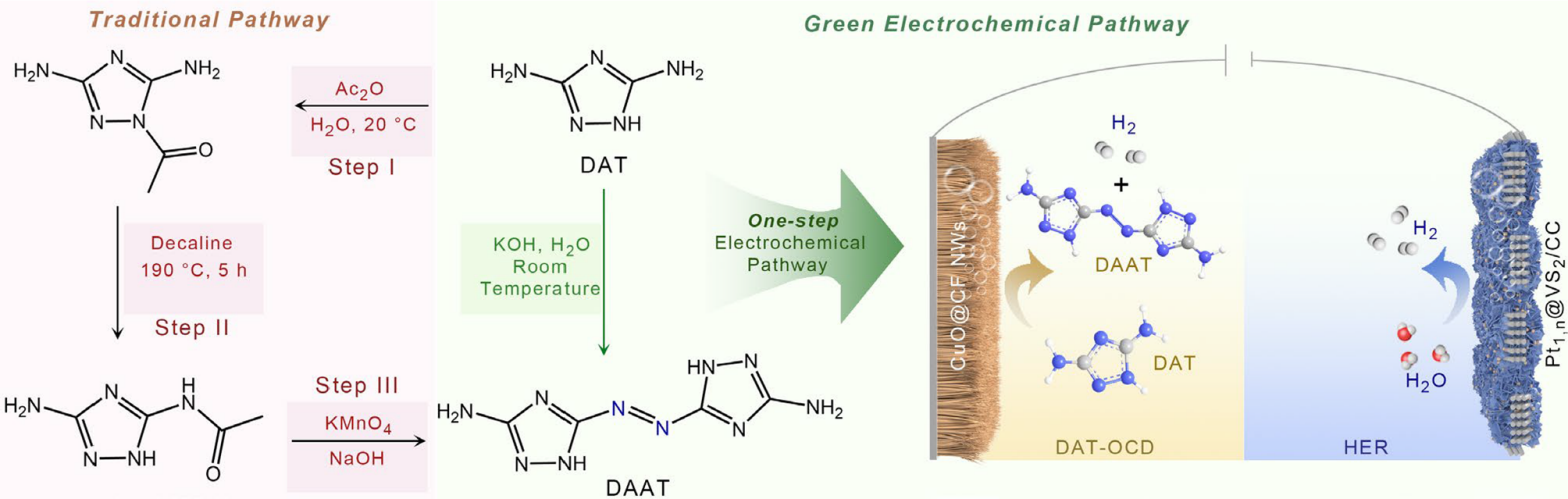
Schematic Illustration of the Traditional Three-Step Pathway to Synthesize DAAT ECs and the One-Step Green Electrochemical Pathway to Synthesize DAAT, Coupled with HER for Low-Energy Value-Added Hydrogen Production

The conventional synthetic process of DAAT usually involves several steps of acylation, thermal isomerization, and azo-bridging (Scheme 1),^29,34^ which is energy-intensive and environmentally harmful. The proposed one-step electrochemical pathway of synthesizing DAAT in an aqueous solution can effectively avoid these drawbacks and improve atom economy, with the added value of the conversion with bipolar hydrogen production.

Moreover, the N–N oxidative coupling to azo bonds are thermodynamically more favorable (around 0.5 V vs RHE) than OER (1.23 V vs RHE), which is a much more desirable alternative to OER in terms of voltage efficiency and selectivity engineering.^35,36^ The resultant hydrogen intermediates may be directly recombined to hydrogen gas via the Tafel mechanism (*H + *H → H_2_) rather than the Volmer oxidation (*H + OH^–^ → * + H_2_O + e^–^) at a relatively low potential.^4^ Based on this concept, we proposed the replacement of the OER with DAT-OCD, which is then coupled with HER to create a hybrid water electrolysis system (Scheme 1). The integrated system is expected to generate hydrogen gas from both the anode and the cathode, along with the value-added DAAT compound at a relatively low electricity power input.

To realize all of the benefits, catalyst design is essential for reducing the overpotential and increasing the energy conversion efficiency of water electrolysis. Single-atom catalysts (SACs) play a crucial role in significantly increasing the atomic utilization efficiency, but are often limited by an insufficient number and inadequate type of active centers, as well as the lack of neighboring sites for catalyzing multistep/electron reactions.^37−39^ Particularly, alkaline HER involves water dissociation (Volmer) and hydrogen recombination (Tafel/Heyrovsky) steps. The SACs still suffer from low active site densities, usually leading to unsatisfactory activity for multistep alkaline HER.^39,40^ Introducing nanoclusters (NCs) into SACs has been demonstrated as an efficient method for providing abundant cooperative active sites for alkaline HER.^41−43^ Currently, SACs/NCs dispersed on various carbon-based substrates have emerged as mainstream heterogeneous catalysts.^44−47^ Compared with carbon-based substrates, the transition metals in transition metal dichalcogenides (TMDs) provide more versatile and flexible coordination environments to regulate the catalytic activity of alkaline HER.^48^

**Figure 1:**
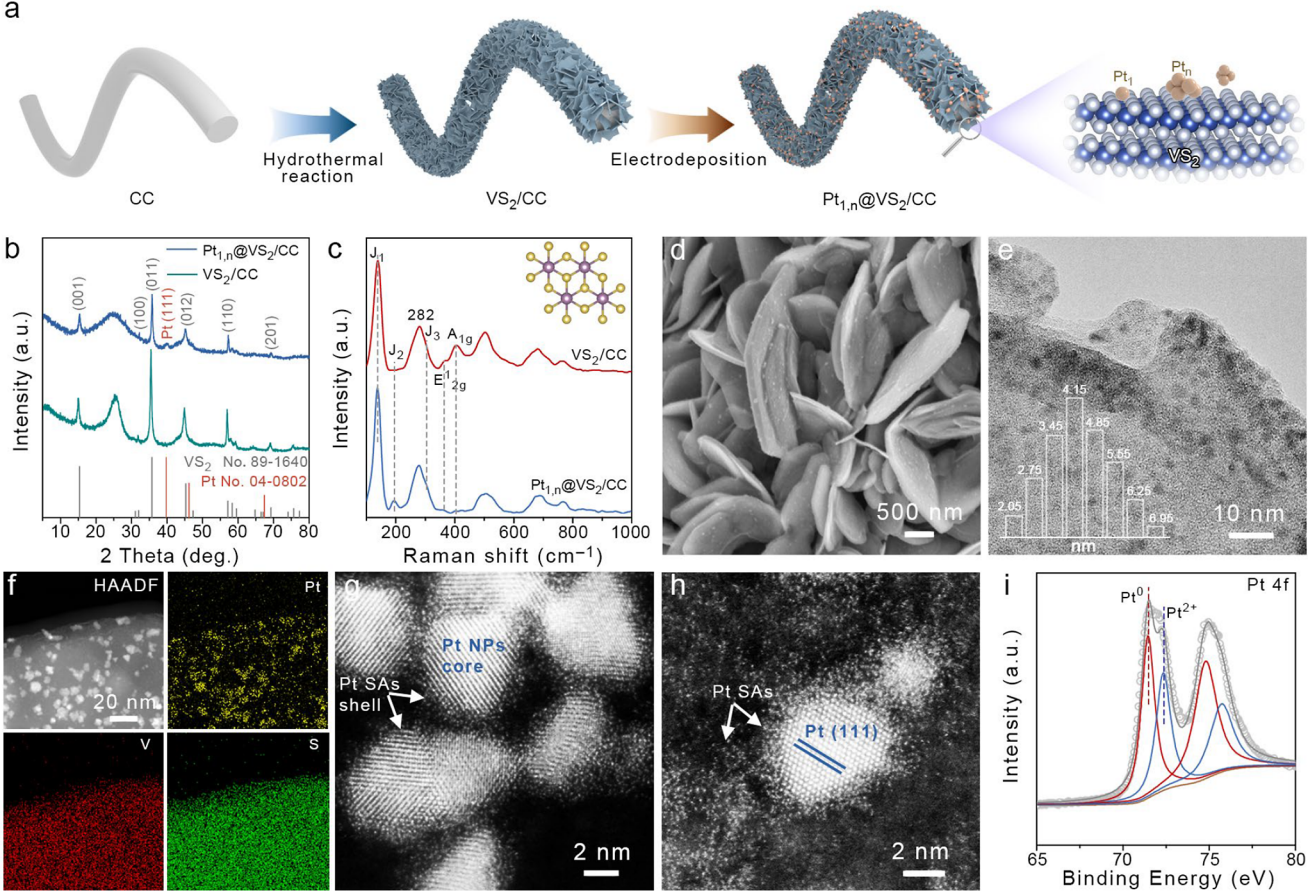
(a) Schematic illustration of the synthesis procedures for Pt_1,n_@VS_2_/CC. (b,c) XRD patterns and Raman spectra of Pt_1,n_@VS_2_/CC and VS_2_/CC. (d) SEM, (e) TEM, and (f) HAADF-STEM images of Pt_1,n_@VS_2_. Inset of (e): size distribution diagram of Pt NPs. (g,h) AC-HAADF-STEM images of Pt_1,n_@VS_2_. (i) High-resolution XPS spectrum of the Pt 4f signal for Pt_1,n_@VS_2_/CC.

**Figure 2:**
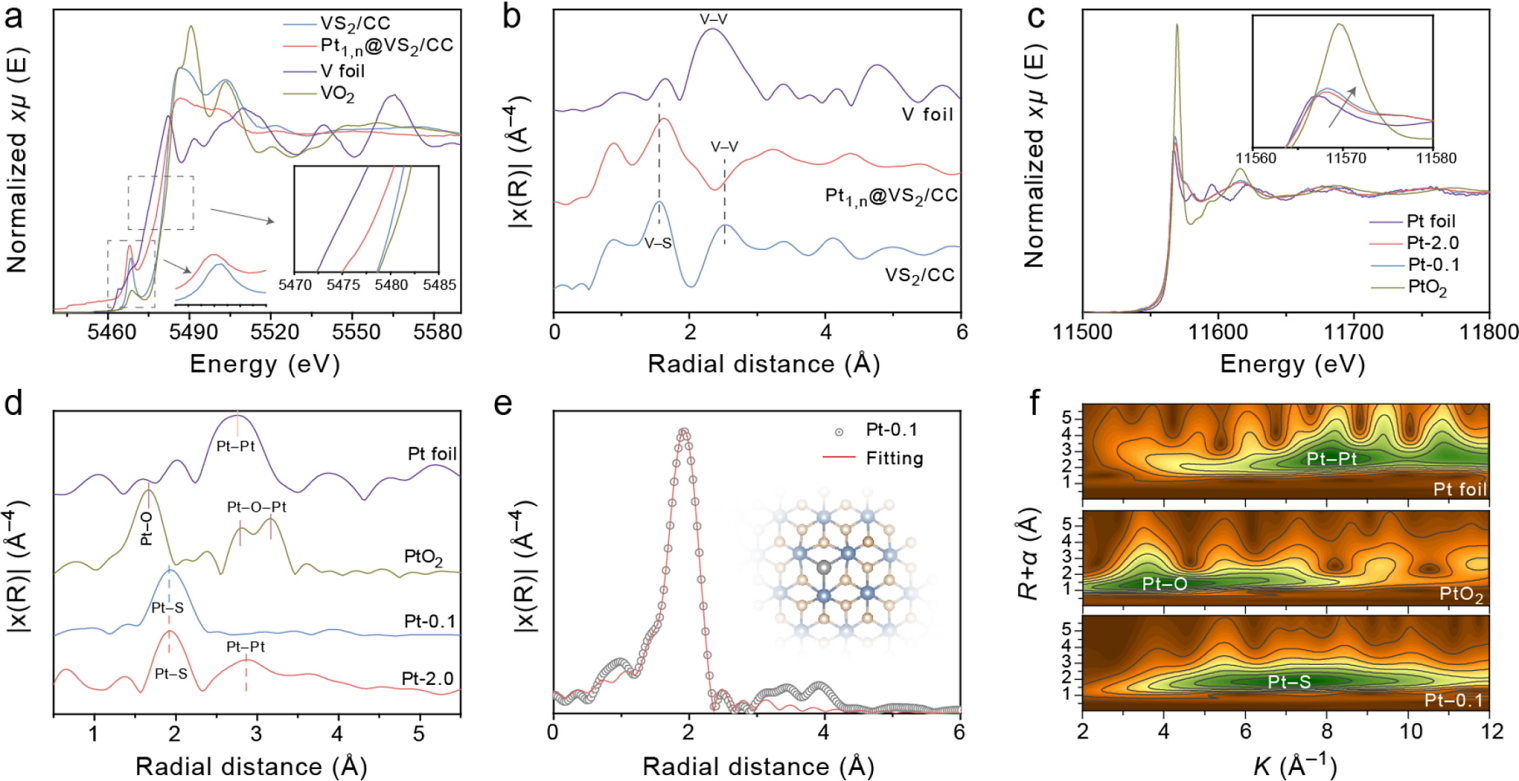
(a) Normalized XANES at the V *K*-edge for VS_2_/CC, Pt_1,n_@VS_2_/CC, and reference samples. (b) *k*^3^-weighted FT-EXAFS of the V *K*-edge for the samples. (c) Normalized XANES at the Pt *L*_*3*_-edge for Pt-0.1, Pt-2.0, and the reference samples. (d) *k*^3^-weighted FT-EXAFS of Pt *L*_*3*_-edge for the samples. (e) Fitting curve of FT-EXAFS of the Pt *L*_*3*_-edge for Pt-0.1. (f) Wavelet transformation (WT) for the FT-EXAFS of the Pt *L*_3_-edge for Pt-0.1.

**Figure 3:**
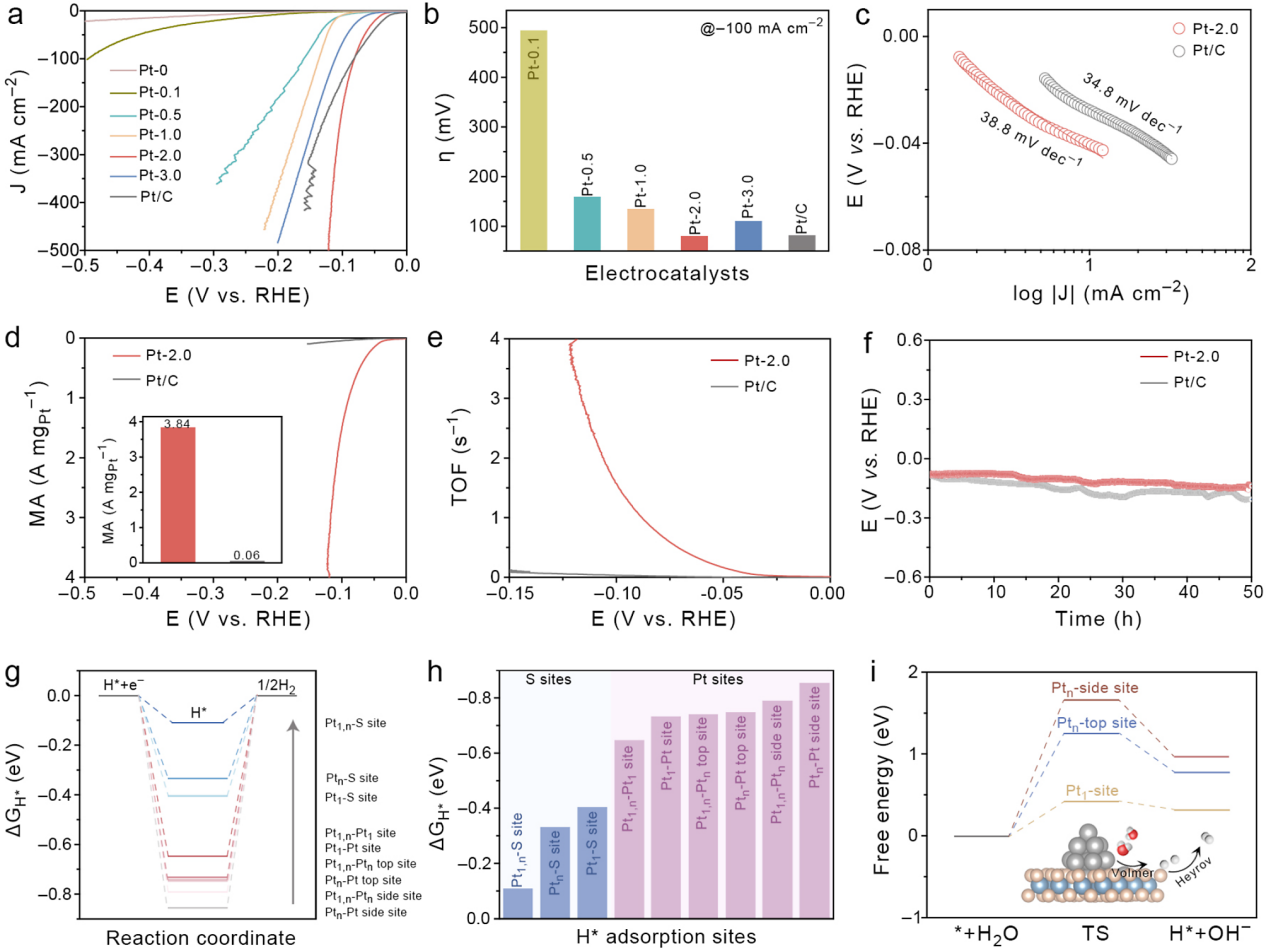
(a) LSV curves of Pt_1,n_@VS_2_/CC with different Pt^4+^ contents. (b) Comparison of the overpotential at −100 mA cm^–2^ for different Pt_1,n_ loadings on VS_2_/CC. (c) Tafel plots of Pt-2.0 and commercial Pt/C. (d,e) MS and TOF plots of Pt-2.0 and commercial Pt/C. (f) Long-term stability of Pt-2.0 and Pt/C at a current density of −100 mA cm^–2^. (g) Calculated Δ*G*_H*_ values at various sites of Pt_1,n_@VS_2_. (h) Comparison of Δ*G*_H*_ on the S and Pt sites of Pt_1_, Pt_n_, and Pt_1,n_. (i) Free energy of the water dissociation barrier on different sites (inset: schematic illustration of the proposed alkaline HER mechanism).

**Figure 4:**
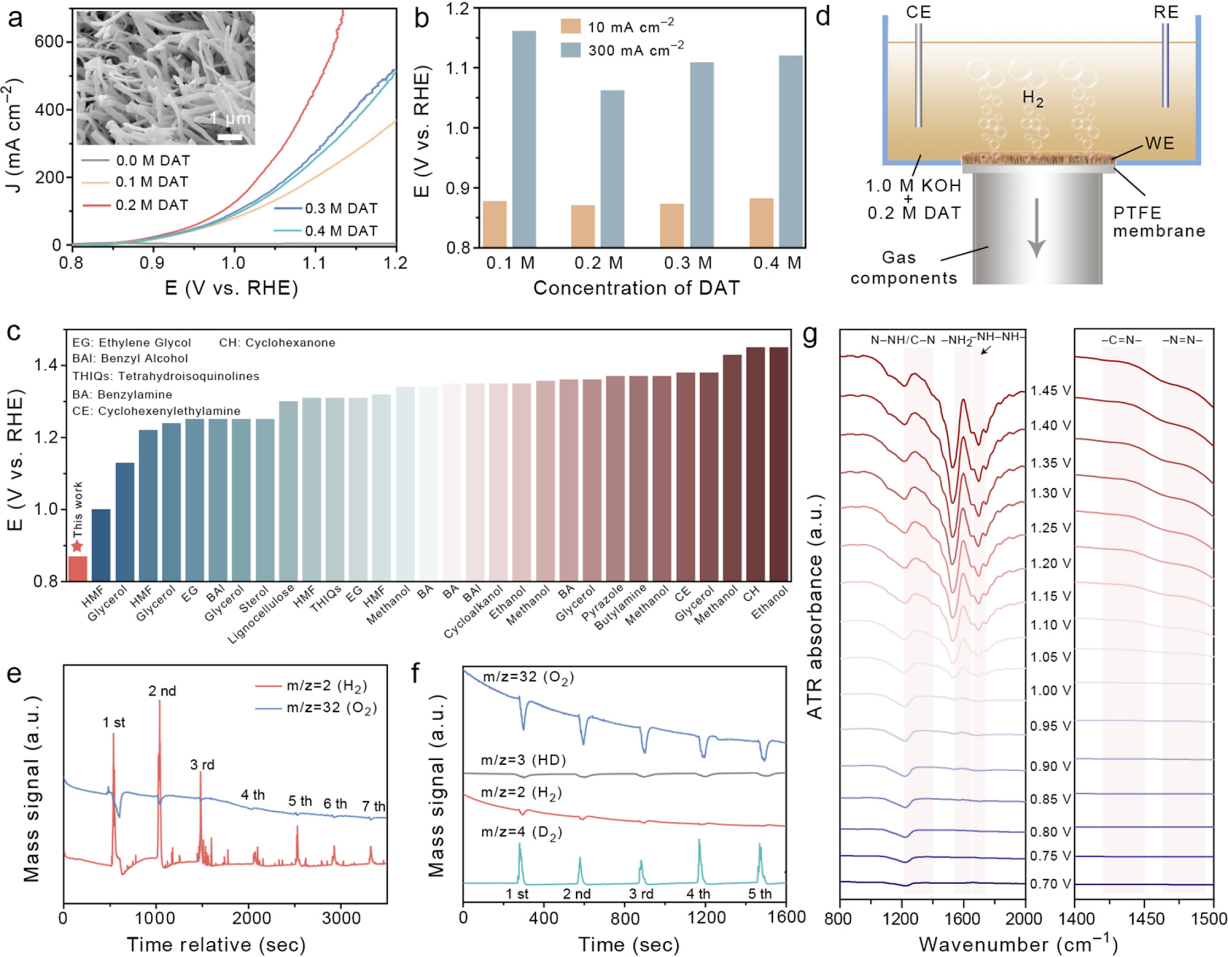
(a) LSV curves of DAT-OCD on CuO@CF NWs in various concentrations of DAT substrates. Inset: SEM image of CuO@CF NWs. (b) Bar graph of the collected potentials of DAT-OCD at the current densities of 10 and 300 mA cm^–2^ at different DAT concentrations. (c) Comparison of DAT-OCD potentials with the reported mainstream upgradation oxidation reactions to produce value-added chemicals at a current density of 10 mA cm^–2^. (d) Schematic illustration of a DEMS reactor during the DEMS tests. (e) In situ DEMS tests of DAT-OCD for seven LSV cycles over specified potential ranges. (f) In situ DEMS tests of the deuterated DAT oxidation reaction for five LSV cycles. (g) In situ ATR-FTIR spectra of DAT-OCD over the specified potential ranges.

Here, platinum single atoms and nanoparticles (Pt_1_ and Pt_n_) were synthesized over metallic vanadium disulfide nanosheets (Pt_1,n_@VS_2_) for the cathodic HER in the hybrid water electrolysis system. As the Group 5 metal disulfide, VS_2_ shows an activated basal plane with intrinsically metallic features due to its relatively low unoccupied states (ε_LUS_ < −5.8 eV), which is a key descriptor for selecting basal-plane-active electrocatalysts.^49^ In our electrocatalyst system, the core anchoring Pt_1_ and the neighboring Pt_n_ can synergistically optimize the electronic structure of VS_2_ to promote the HER activity, even superior to the commercial Pt/C, to reach a high current density (>100 mA cm^–2^). The integrated hybrid water electrolysis system of DAT-OCD and HER showed ultralow cell voltages of 0.946 and 1.129 V for bipolar hydrogen production to reach 10 and 100 mA cm^–2^, respectively, and delivered a low electricity consumption of 1.3 kWh per m^3^ H_2_ at an evaluated current density of 100 mA cm^–2^. Meanwhile, the value-added DAAT solid compound was successfully generated on the anode side. The assembled flow device of the hybrid electrolysis system maintained stable catalysis over 300 h at 500 mA cm^–2^, revealing its excellent tolerance and stability for industrial hydrogen production and value-added chemical cogeneration. This study paves a promising avenue for energy-saving bipolar hydrogen production and environmentally friendly synthesis of azo-triazole chemicals (Figures 1–4).

## Results and Discussion

### Synthesis and Characterization of Cathodic HER Catalysts

Before the assembly of the hybrid water electrolysis system with DAT-OCD and HER, the cathodic and anodic electrocatalysts were first designed. The Pt_1,n_@VS_2_ supported on conductive carbon cloth (Pt_1,n_@VS_2_/CC) as the cathodic HER catalysts was prepared by a two-step process. The procedures involve a hydrothermal reaction to support VS_2_ nanosheets (VS_2_ NSs) on the CC substrate followed by electrodeposition of Pt NPs and Pt SAs on VS_2_ NSs (Figure 1a).

Scanning electron microscopy (SEM) shows a vertical and staggered alignment of the VS_2_ NSs on the CC substrate (Figure S1). Transmission electron microscopy (TEM) and high-resolution TEM (HRTEM) show that the lattice distance is 0.278 nm, corresponding to the (100) crystal plane of VS_2_ (Figure S2a,b). High-angle annular dark-field scanning transmission electron microscopy (HAADF-STEM) and elemental mappings of VS_2_ are performed to reveal the homogeneous distribution of V and S on the VS_2_ NSs (Figure S2c–e). X-ray diffraction (XRD) patterns indicate that all of the diffraction peaks are assigned to the (001), (100), (011), (012), (110), and (201) planes of 1T-type VS_2_ (JCPDS No. 89-1640).^50^ After the deposition of Pt_1,n_, the main diffraction peaks of Pt_1,n_@VS_2_/CC are almost identical to those of VS_2_/CC with only a small new peak located at ∼39.7°, which may be ascribed to the Pt (111) plane (JCPDS No. 04-0802).^41^ This result indicates that the electrodeposition of Pt_1,n_ did not change the structure of the VS_2_ substrate, and only a trace amount of Pt_1,n_ is loaded over VS_2_ (Figure 1b).

The inductively coupled plasma results confirm that the contents of Pt and V in Pt_1,n_@VS_2_ are 0.128 mg_Pt_ cm^–2^ and 1.22 mg_V_ cm^–2^. The Raman spectra further verified the crystal structure of Pt_1,n_@VS_2_. As shown in Figure 1c, the characteristic vibration bands of VS_2_/CC at approximately 141, 282, 304, 367, and 406 cm^–1^ are assigned to the *J*_1_, *E*_g_, *J*_3_, *E*^1^_2g_, and *A*_1g_ vibrational modes of the octahedral (1T) phase of VS_2_, respectively, with some trigonal prismatic (2H) phase of VS_2_ coexisting in the samples.^51^ Interestingly, the deposition of Pt_1,n_ (if there is no special illustration, Pt_1,n_@VS_2_/CC is indicated as Pt-2.0 with the optimal Pt ion content of 2.0 mL; the detailed influence of the Pt ion concentration on the HER performance is shown in Figure 4) induces the disappearance of the *A*_1g_ mode and is accompanied by a fresh peak at ∼195 cm^–1^ corresponding to the *J*_2_ mode of 1T-VS_2_. This result may be due to the transformation of 2H to 1T-VS_2_ during the electrodeposition of Pt_1,n_. We speculate that such a special electrochemical reduction environment could introduce S-vacancies and inject electrons to the S–V–S framework, and the phase transformation is triggered.^52,53^

The sulfur vacancies in Pt_1,n_@VS_2_/CC are further supported by electron paramagnetic resonance (EPR). Compared with VS_2_/CC, a strong paramagnetic absorption signal is observed at *g* = 2.001 for Pt_1,n_@VS_2_/CC, referring to the unpaired electrons being trapped in sulfur vacancies (Figure S3).^54^ After the electrodeposition of Pt_1,n_ on VS_2_/CC, the morphological changes of nanosheets are not observed in the SEM image. Some nanoparticles are homogeneously dispersed on the nanosheets, indicating the successful loading of Pt_1,n_ on VS_2_/CC (Figures 1d and S4). Although in the HRTEM image of Pt_1,n_@VS_2_, small dark nanoparticles with an average diameter of 4.31 nm are uniformly dispersed on the VS_2_ NSs, the corresponding HAADF-STEM images, EDS spectra, and elemental mappings indicate that these are Pt NPs on the VS_2_ surface (Figures 1e,f and S5). However, further information on Pt on VS_2_ NSs can be collected by AC HAADF-STEM, where the Pt NPs (core) and Pt SAs (shell) coexist to form a novel core–shell structure (Figure 1g,h). The lattice distance of Pt NPs (0.226 nm) is indicative of the Pt (111) plane. The coexistence of Pt NPs and Pt SAs can provide multiple types of active sites for better promoting multistep kinetic reactions.^39^

X-ray photoelectron spectroscopy (XPS) results of the Pt 4f signal for Pt_1,n_@VS_2_/CC show two pairs of peaks located at 71.4/74.8 and 72.3/75.7 eV, respectively. The former can be attributed to metallic Pt^0^, while the latter corresponds to Pt^2+^ (Figure 1i).^55^ This result supports the coexistence of the dominant Pt NPs and Pt SAs in VS_2_/Pt_1,n_. High-resolution XPS of V 2p and S 2p signals of the samples is carried out to uncover the electronic interaction of VS_2_ and Pt_1,n_. The V 2p spectra of VS_2_/CC fitted with four peaks at 513.7, 521.2, 516.9, and 524.2 eV are assigned to V^2+^ 2p_3/2_, V^2+^ 2p_1/2_, V^4+^ 2p_3/2_, and V^4+^ 2p_1/2_, respectively.^56^ After the deposition of Pt_1,n_, the peaks shifted to a lower binding energy (*ca*. 0.15 eV), indicating that the loading of Pt_1,n_ induces electron accumulation around V, and more metallic V with 1T-VS_2_ are formed (Figure S6a). The same trend is also observed at the S 2p signal (Figure S6b). High-resolution S 2p XPS spectra of VS_2_/CC show four spin–orbit peaks at 160.9/162.1 and 163.1/164.3 eV, ascribing to S^2–^ species and metal–sulfur excitation in VS_2_, respectively.^57^ Notably, the S^2–^ ratio in Pt_1,n_@VS_2_ is lower than that of VS_2_, followed by a binding energy shift to a lower position (ca. 0.15 eV), which may be due to the electrochemical reduction conditions during the deposition of Pt_1,n_ and the strong metal–support electronic interaction between Pt_1,n_ and VS_2_. The electron reconfiguration in Pt_1,n_@VS_2_ is able to regulate the adsorption strength of alkaline HER intermediates for promoting the catalytic activity.

X-ray adsorption spectroscopy was performed for further exploration of the local coordination environment and the electronic structure of Pt_1,n_@VS_2_. As shown in Figure 2a, the V *K*-edge X-ray absorption near-edge spectra (XANES) of VS_2_/CC is similar to those of the VO_2_ reference sample, indicating that the V atoms exhibit an oxidation state of +4. However, the deposition of Pt_1,n_ induces a valence of V in VS_2_ lower than +4, as evidenced by the location of the V *K*-edge XANES of Pt_1,n_@VS_2_/CC between VO_2_ and V foil. The results further suggest that the loading of Pt_1,n_ on VS_2_ leads to electron transfer from Pt_1,n_ to VS_2_, which is in line with the XPS results. In addition, the intensity of pre-edge peak (1*s* → 3*d*) of Pt_1,n_@VS_2_/CC increases compared with VS_2_/CC, suggesting the phase conversion of 2H into the 1T phase of VS_2_, which is in concert with the Raman result.^58^

In the V *K*-edge Fourier transform extended X-ray absorption fine structure (FT-EXAFS) spectra, the peaks at 1.56 and 2.55 Å are ascribed to the shell coordination of V–S and V–V bonds for VS_2_/CC, respectively (Figure 2b).^59^ Note that the multiple scattering of V–S peaks shift to a larger distance after the loading of Pt_1,n_ on VS_2_, resulting from the lattice tensile strain and breaking of the V–S bond to create abundant unsaturated coordinated S caused by the immobilization of Pt_1,n_.^60,61^ However, the second-shell V–V intensity of Pt_1,n_@VS_2_ shows an apparent decrease, suggesting the occurrence of structural disorder induced by atomic rearrangement after the doping of Pt_1,n_.^61,62^ The normalized Pt *L*_3_-edge XANES spectra of Pt-0.1 (the content of Pt ions in the electrolyte is 0.1 mL) and Pt-2.0 are shown in Figure 2c, and the intensities of the white line for Pt-0.1 and Pt-2.0 are located between the Pt foil and the PtO_2_ reference samples, indicating that Pt atoms carry positive charges. However, the higher white-line intensity of Pt-0.1 and more positive edge shift of Pt-0.1 compared to that of Pt-2.0 are attributed to the higher content of positively charged Pt SAs in Pt-0.1. The ratio of Pt SAs to Pt NPs can be well controlled by adjusting the concentration of the Pt^4+^ solution before the electrodeposition of Pt_1,n_ on VS_2_. The formation of Pt SAs and Pt NPs in Pt_1,n_ is further proved by the Pt *L*_3_-edge FT-EXAFS spectra (Figure 2d). Pt-0.1 exhibits only one dominant peak located at 1.93 Å, assigned to Pt–S bonding, proving the single atomic dispersed form of Pt on VS_2_ under a low concentration of Pt^4+^. In addition to the Pt–S bond, the new peak centered at 2.88 Å in Pt-2.0 corresponds to the Pt–Pt bond. This result indicates that the increased concentration of the Pt^4+^ precursor could induce partial aggregation into nanoparticles and lead to the coexistence of Pt SAs and Pt NPs in Pt_1,n_.

The EXAFS spectrum of Pt-0.1 was further treated by least-squares EXAFS curve-fitting analysis (Figure 2e). The average coordination numbers of Pt–S and Pt–V bonds are estimated to be 3.1 and 3.0. We can thus speculate that the Pt atoms may be immobilized in the S vacancy and coordinated with three V and S atoms (Table S1). The coordination environment of Pt in Pt-0.1 is then verified by wavelet transform (WT) analysis (Figure 2f). The WT reveals that the Pt bonding location can be speculated based on the spectral maximum intensity of the Pt–O bond in PtO_2_ (*k* ≈ 3.98 Å^–1^) and the Pt–Pt bond in the Pt foil (*k* ≈ 8.09 Å^–1^). The result indicates that the weight of coordination elements is located between Pt and O such as S (*k* ≈ 7.19 Å^–1^), further supporting the Pt–S bonds in Pt-0.1.

### Alkaline HER Tests and Cathodic Density Functional Theory (DFT) Calculations

The cathodic HER performance of the prepared Pt_1,n_@VS_2_/CC is the key reaction for an energy-efficient hybrid water electrolysis system. The influence of the Pt^4+^ concentration on the HER performance is first investigated by linear sweep voltammetry (LSV), and the corresponding overpotentials at a current density of −100 mA cm^–2^ (η_100_) are recorded (Figure 3a,b). A notably lower η_100_ is obtained over the Pt-2.0 (81.3 mV) than those of Pt-0.1 (495 mV), Pt-0.5 (160 mV), Pt-1.0 (136 mV), Pt-3.0 (11.1 mV), and commercial Pt/C (81.9 mV). Under a current density of −10 mA cm^–2^, Pt-2.0 still showed a low overpotential (η_10_) of 40.3 mV, which is slightly higher than that of the commercial Pt/C (27.8 mV). However, Pt-2.0 delivers a steady current enhancement to a high current density (>100 mA cm^–2^) with a gradually lower overpotential than that of the Pt/C.

Compared with the sharp fluctuation of the LSV curve under a high current density of Pt/C, a rather stable flat curve is noted for Pt-2.0 under the same conditions, which may be attributed to the 3D self-supporting Pt_1,n_@VS_2_/CC nanosheet architecture, with the superhydrophilic surfaces allowing for faster bubble removal during HER.^63^ The superhydrophilicity of Pt-2.0 is proven by the contact angle measurement (Figure S7). Compared with the CC substrate, the support of VS_2_ and VS_2_/Pt_1,n_ increases the surface wettability with a contact angle of 0°, and the water droplet seeps through the entire matrix immediately, suggesting the superhydrophilic feature of the electrocatalyst. Such a unique nature could provide a more wettable surface to expel the generated H_2_ gas bubbles and improve the cycling stability.

Tafel plots are obtained to determine the kinetic rate and possible mechanism of alkaline HER (Figure 3c). The Tafel slope of Pt-0 (VS_2_/CC) is measured to be 142.8 mV dec^–1^, indicating that the rate-determining step (RDS) of the Volmer process dominates the reaction. However, the loading of Pt_1,n_ on VS_2_/CC shows a significant decrease in the Tafel slope to 38.8 mV dec^–1^, which is close to that of the commercial Pt/C (34.8 mV dec^–1^). This result suggests the Volmer–Heyrovsky pathway, with the electrochemical H_2_ desorption on Pt_1,n_@VS_2_/CC as the RDS.^64^ It also reveals that the anchoring of Pt_1,n_ sites may assist in reducing the energy barrier of the Volmer step to promote water dissociation and produce H* for the following Heyrovsky step; the speculation is further supported by DFT calculations, as discussed below. As both Pt SAs and Pt NPs coexist in Pt_1,n_, potassium thiocyanate (KSCN) and ethylenediaminetetraacetic acid (EDTA) are introduced in the electrolyte, respectively. EDTA can block the single-atom site by coordination, while SCN^–^ deactivates both single atoms and nanoparticles by covering the surface of Pt_1,n_.^65^ As shown in Figure S8, the η_10_ of Pt_1,n_@VS_2_/CC delivers a negative shift by ca. 55 mV after the introduction of EDTA, while the η_10_ increases by only *ca*. 10 mV after the addition of KSCN. The poisoning results suggest that although Pt SAs and Pt NPs both deliver a highly efficient alkaline HER activity, Pt SAs play a dominant role.

The intrinsic activity and atomic utilization of Pt_1,n_ are then evaluated by mass activity (MS) tests. Pt-2.0 exhibits an MS value of 1.55 A mg_Pt_^–1^ at an overpotential of 100 mV, which is about 35 times higher than that of commercial Pt/C (0.04 A mg_Pt_^–1^) (Figure 3d). The turnover frequency (TOF) was determined to further evaluate the intrinsic activity of the catalysts. Pt-2.0 reaches a high TOF value of 1.57 s^–1^ at an overpotential of 100 mV, largely surpassing commercial Pt/C (0.04 s^–1^, ∼34.5 times) (Figure 3e). Stability is another important factor that determines the lifetime of catalysts (Figure 3f). A catalyst should be operated at high current densities to meet the industrial demand. As a sharp comparison of Pt/C, the long-term stability test of the elaborate Pt-2.0 is performed over 50 h, which shows negligible decay of activity to reach a current density of −100 mA cm^–2^.

DFT calculations were then performed to reveal the synergistic effect among the VS_2_ substrate, Pt SAs, and Pt NPs. The structures of Pt_1_ (Pt SA is located at the S vacancy of the VS_2_ (002) plane), Pt_n_ (Pt_13_ NCs load on the VS_2_ (002) plane), and Pt_1,n_ (coexistence of Pt_1_ and Pt_n_) were constructed and then fully relaxed (Figure S9). All of the possible Pt or S active sites are considered in the H* adsorption free energy (Δ*G*_H*_) calculation. As a rule of thumb, an ideal HER catalyst delivers a thermoneutral value of Δ*G*_H*_ close to zero.^66^Figure 3g displays the Δ*G*_H*_ on various Pt and S sites of Pt_1_, Pt_n_, and Pt_1,n_, and the corresponding Δ*G*_H*_ values are collected and shown in Figure 3h (Figure S10). It is clear that the S site exhibits thermodynamics more favorable for H* adsorption than those of Pt sites. Especially, compared with the Δ*G*_H*_ on S sites adjacent to Pt_n_ (−0.33 eV) or Pt_1_ (−0.40 eV), the H* adsorption on S sites adjacent to Pt_1,n_ shows the most favorable Δ*G*_H*_ of −0.10 eV. This result indicates that the electron interactions between Pt_1_ and Pt_n_ could modulate charge redistribution and achieve optimal binding strengths of H* on S active sites of Pt_1,n_. To further clarify the specific active sites for hydrogen evolution, in situ surface-enhanced Raman spectroscopy (SERS) was performed across a potential range of 0 to −0.8 V vs. RHE (Figure S11a). When the potential became more negative, a Raman peak located at ca. 2580 cm^–1^ gradually appeared, and its intensity increased as the potential was further reduced to −0.8 V vs. RHE. Such a peak was ascribed to the stretching vibration of the S–H bond.^67−69^ However, the Pt–H vibration located around 1990–2100 cm^–1^ does not exist, suggesting that the active sites for hydrogen formation are mainly contributed by the S sites in Pt_1,n_@VS_2_.^70,71^ To further analyze the variation of the H* binding capacity with the S sites in Pt_1,n_@VS_2_, Pt_1_@VS_2_, and Pt_n_@VS_2_, we calculated the *p*-band centers (ε_p_) of active S sites for H* adsorption since the H* adsorption capacity on the S sites of sulfides is closely related to the *p* electron states near the Fermi level.^72^ As shown in Figure S11b, compared with Pt_1_@VS_2_ and Pt_n_@VS_2_, the ε_p_ of S on the Pt_1,n_@VS_2_ is further away from the Fermi level, indicating the electronic interaction of Pt SAs and Pt NPs jointly regulating the adsorption of H* on S sites, close to thermoneutrality. This result was in agreement with the Δ*G*_H*_ discussed above.^73^ On the other hand, the thermodynamic trends for water molecule dissociation of the Volmer step are critical to ensure the fast alkaline HER process. The energy barriers of the Volmer step on Pt_1_ and Pt_n_ sites are calculated (Figures 3i and S12). The Pt_1_ site meets a lower transition state energy of 0.43 eV compared to the Pt_n_-top (1.26 eV) and Pt_n_-side (1.67 eV) sites, indicating that the water molecules are more likely to dissociate on the Pt SA site. In addition, the weak bonding energy of the H_2_O molecule on the S site of Pt_1,n_ (0.93 eV) suggests that the S sites may not be the active sites for water dissociation (Figure S13). These results mean that Pt SAs are responsible for the decrease of the Tafel slope via the promotion of the Volmer step (water dissociation) after the loading of Pt_1,n_, which is in agreement with the Pt ion concentration gradient and poison tests. DFT calculations revealed the cascade catalysis of alkaline HER and that the Pt SAs site promotes the Volmer step, and the produced H* spill over to the S sites via the Heyrovsky pathway for fast hydrogen production.

### Anodic Electrochemical DAT-OCD Investigation

The high thermodynamic potential of the OER impels us to explore thermodynamically favorable and value-added alternative oxidation reactions for the reduction of energy consumption in water electrolysis systems. However, the key challenge of hybrid water electrolysis systems is the market-size mismatch between cathodic hydrogen production and the anodic oxidation product for large-scale deployment. The bipolar hydrogen production strategy-enabled hydrogen production at both the anode and the cathode could well overcome this challenge.^4^ With this consideration in mind, we started investigating a thermodynamically favorable N–N oxidative coupling reaction of amino compounds to synthesize value-added azo chemicals. The CuO nanowire supported on 3D copper foam (CuO@CF NWs) was used as a model catalyst for DAT-OCD as the CuO-based catalysts are inert for competitive OER and active for organic small-molecule oxidation.^74^

CuO@CF was prepared by the calcination of Cu(OH)_2_@CF NWs, obtained from in situ etching in alkaline liquor (Figure S14). The XRD pattern verifies the pure CuO supported on CF substrates (Figure S15). The DAT-OCD activities of CuO@CF and other Cu- or Ni-based benchmark catalysts are compared using LSV tests in 1.0 M KOH containing optimal 0.2 M DAT (Figures 4a and S16). The onset potential of DAT-OCD on CuO@CF is as low as 0.83 V vs RHE and delivers 0.87 and 1.06 V vs RHE to reach current densities of 10 and 300 mA cm^–2^, respectively. These results are much lower than those of copper foam (1.21 V), Cu(OH)_2_@CF NWs (1.38 V), and nickel foam (not up to the target current density) at 300 mA cm^–2^, and the commercial Pt wire delivered a relatively low DAT-OCD activity.

In view of the low activity of the competing OER reaction and the excellent performance of the oxidation of organic small molecules on Cu-based catalysts, the emphasis was on the Cu-based catalysis for DAT-OCD.^4^ The advantages of CuO@CF over other Cu-based catalysts were further studied by in situ attenuated total reflection Fourier transform infrared (ATR-FTIR) spectroscopy. In situ ATR-FTIR of DAT-OCD for CuO@CF, Cu(OH)_2_@CF, and bare CF were comparatively assessed to evaluate the adsorption characteristics of the DAT substrate on Cu-based electrocatalysts so as to further clarify the advantages of DAT-OCD on the CuO@CF electrode (Figure S17). The characteristic peak at ∼1593 cm^–1^, due to the stretching vibration of the −NH_2_ group in DAT, represents the adsorbed DAT molecules on the catalysts.^75^ The increased peak intensities can be attributed to a high density of the adsorbed substrate molecules on the catalyst surface, reflecting the strengthened adsorption capacity.^76,77^ Cu(OH)_2_@CF and the bare CF support showed a relatively weak adsorption peak of −NH_2_, indicating a weak adsorption behavior for the DAT substrate. In contrast, a more pronounced peak at ∼1593 cm^–1^ was detected on CuO@CF, indicating the strong adsorption therein. In addition, the adsorption capacity of OH* on Cu-based catalysts was evaluated by in situ ATR-FTIR since the reaction of DAT with OH* is crucial for subsequent dehydrogenation steps in DAT-OCD. As shown in Figure S18a, two peaks at ∼3702 and ∼3784 cm^–1^ during DATOR on CuO@CF can be assigned to the stretching vibrations of O–H.^78,79^ The peak intensity becomes more pronounced with increasing applied potential. In contrast, the peak intensity of OH* was much weaker during DAT-OCD on bare CF and Cu(OH)_2_@CF (Figure S18b,c). These results imply an enhanced interaction between OH* and the CuO@CF catalyst for promoting the dehydrogenation of DAT, compared with Cu(OH)_2_@CF and bare CF. The above results indicate the superior activity of DAT-OCD on CuO@CF, relative to those on Cu(OH)_2_@CF and CF.

The DAT-OCD activity of CuO@CF NWs under various DAT concentrations (0.1, 0.2, 0.3, 0.4 M) shows a volcano trend that the 0.2 M DAT delivers the highest activity at 10 mA cm^–2^ and high current density (300 mA cm^–2^) (Figure 4b). The LSV curves also exhibit that the DAT-OCD could reach an industrial-level current density >500 mA cm^–2^, revealing the potential for incorporating DAT-OCD in the anode of the hybrid water electrolysis system. The DAT-OCD activity with an ultralow working potential is superior to those of the state-of-the-art upgrading oxidation reactions, such as 5-hydroxymethylfurfural oxidation reaction,^80^ glycerol oxidation,^81^ methanol oxidation,^82^ and ethanol oxidation^83^ (Figure 4c). The optimal DAT concentration (0.2 M) was then used in subsequent DAT-OCD tests. Considering the low oxidation potential of DAT-OCD and the presence of reactive hydrogen atoms in the amino functional group of DAT, we postulate that the adsorbed hydrogen atom (H*) originating from the amino groups of DAT may undergo a Tafel recombination (H* + H* → H_2_) to form H_2_ rather than the large-kinetic-barrier Volmer pathway (H* + OH^–^ → H_2_O + e^–^).^4,12^

As a proof-of-concept, in situ differential electrochemical mass spectrometry (DEMS) was performed to quantify the amount of produced gas during DAT-OCD. As shown in Figure 4d, a three-electrode reaction tank was set up to perform the in situ DEMS, and the CuO@CF NWs as the working electrode was tiled on the porous Teflon membrane. During the reaction, the gas components were pumped through the membrane and collected by DEMS for the fast detection of gas products. Before the DAT-OCD tests, a control experiment of the OER test in 1.0 M KOH without the addition of DAT was carried out using LSV over the potential range of 0.7–2.2 V vs RHE for three cycles (Figure S19a). As expected, the mass signal for *m*/*z* = 32 (O_2_) was detected at 1.83 V, proving the poor OER activity of CuO@CF. In addition, the mass signal for *m*/*z* = 2 (H_2_) was not detected, indicating that the hydrogen atoms in H_2_O are not involved in the anodic H_2_ evolution (Figure S19b). Then, the DEMS was performed in 1.0 M KOH + 0.2 M DAT over the potential range of 0.7–2.2 V vs RHE for the first two cycles (Figure 4e). Interestingly, the signal of *m*/*z* = 2 (H_2_) shows an intense peak accompanied by strong fluctuation with numerous H_2_ bubbles observed (Video S1), while the signal of *m*/*z* = 32 (O_2_) was not detected even when the potential was shifted to the OER range (>1.83 V vs RHE). The cutoff potentials are then controlled to 1.4, 1.3, and 1.4 V for the third, fourth, and fifth to seventh cycles, respectively, to adjust the amount of H_2_. This result indicates the high correlation between the applied potential and the amount of H_2_: the higher the applied potential, the higher the amount of produced H_2_. Meanwhile, the competitive OER could be well inhibited to promote the Faradaic efficiency of DAT-OCD. The origin of H in H_2_ was verified by an isotope labeling experiment.

The active hydrogen of amino in DAT was deuterated by immersing 0.2 M DAT in deuteroxide (Figure S20). The in situ DEMS spectra show that *m*/*z* = 4 (D_2_) of gas products is detected, followed by a weak mass signal of *m*/*z* = 2 (H_2_), which may be due to the incomplete deuteration of amino in DAT. When the concentration of DAT was reduced to 0.1 M, the complete deuteration is supported by ^1^H nuclear magnetic resonance (NMR) as evidenced by the disappearance of the H signal of DAT (Figure S21). As noted from Figure 4f, a strong signal for *m*/*z* = 4 (D_2_) was detected, whereas there were no signals of *m*/*z* = 2 (H_2_), *m*/*z* = 3 (HD), and *m*/*z* = 32 (O_2_), suggesting that all of the D atoms in D_2_ originated from the deuterated amino group in the DAT substrate, with no O_2_ formation in the process. In other words, the competitive OER and Heyrovsky pathway are not involved in the DAT-OCD. Note that the detection of a negative signal of *m*/*z* = 32 is due to the H_2_ fluctuation in the sampled gaseous mixture for the DEMS analysis. Based on the above discussion and in situ ATR-FTIR (Figure S18), it can be demonstrated that the H_2_ production mechanism at the anode is the dehydrogenation of the amino group triggered by OH*, and the produced H* from two DAT molecules recombine to form H_2_ via the Tafel pathway, rather than the Heyrovsky or Volmer step (produce nonvalued H_2_O), leading to satisfactory atom efficiency of the bipolar hydrogen production by anodic N–N oxidatively coupled dehydrogenation and HER.

In situ ATR-FTIR spectroscopy was performed to further verify the dehydrogenation and H_2_ production mechanism of DAT-OCD. The spectra were collected by applying various potentials via chronoamperometry between 0.7 and 1.45 V vs RHE without the competitive OER process (Figure 4g). The large vibration bands at ∼1593 cm^–1^ are attributed to the −NH_2_ groups of the DAT substrate.^75^ It can be seen that the peak of −NH_2_ appears at a potential of 0.85 V vs RHE in accord with the onset potential of DAT-OCD in the LSV test (Figure 4a), indicating that the oxidation of DAT begins at an ultralow potential input than that of OER. The pronounced growth of the −NH_2_ peak with an increase in the applied potential suggests that more DAT substrate was adsorbed on the surface of CuO and promotes the kinetics of DAT-OCD. The vibration bands at ∼1284 cm^–1^ originated from the N–NH/C–N vibration within the triazole rings in DAT.^84^ Meanwhile, we observed weak growth of the peaks at 1435 and 1485 cm^–1^, respectively, assigned to −C=N– and −N=N– vibrations.^75^ The similar peak intensity variation trends of −C=N– and those of −N=N– and −NH–NH– with the increase of potential is attributed to the fast kinetics of DAT-OCD. At a relatively low applied potential, the DAT-OCD reaction did not occur due to kinetic limitations, presenting no apparent signal of the vibration bands. When the applied potential was increased, the DAT-OCD reaction was triggered, and the adsorption signals of −NH_2_ and −C=N– bonds over the DAT substrate were detected. Meanwhile, the formation of −N=N– bonds was also detected due to the fast kinetics of the DAT-OCD reaction. On the other hand, the adsorption of the DAT substrate on the CuO surface was also enhanced with the increasing potential, leading to a similar trend of −C=N– bonding to those of −N=N– and −NH–NH– bonding. In contrast, ATR-FTIR of the OER without the addition of DAT (Figure S22a) shows no apparent peak intensity variations with the increase of the potential, indicating the successful synthesis of DAAT azo products.

Attention was then directed to the DAT-OCD mechanism. There are two main pathways in electrochemical organic oxidation: electrochemical–chemical oxidation and OH* oxidation.^85^ The former involves the reaction of OH^–^ with the catalyst surface to form high-valence derivatives as the active sites, which activate and oxidize the substrate molecules into target products, accompanied by the reduction of the catalyst to its initial state. The latter involves OH^–^ adsorption at a certain potential, forming OH* without the valence change of the catalyst. The OH* then activates nucleophilic groups (e.g., amino) to promote N–H cleavage and produce oxidation products and water molecules. Whether the OH^–^ participated in the DAT-OCD was verified by ATR-FTIR (Figures S18 and S22), where OH* was detected in both DAT-OCD and OER. This result suggests that OH* is involved in the DAT-OCD reaction. Hence, the OH* oxidation mechanism dominates the DAT-OCD reaction. Besides, the upward peaks at 1720 and 1665 cm^–1^ correspond to −NH–NH– coupling bonds.^86^

**Figure 5:**
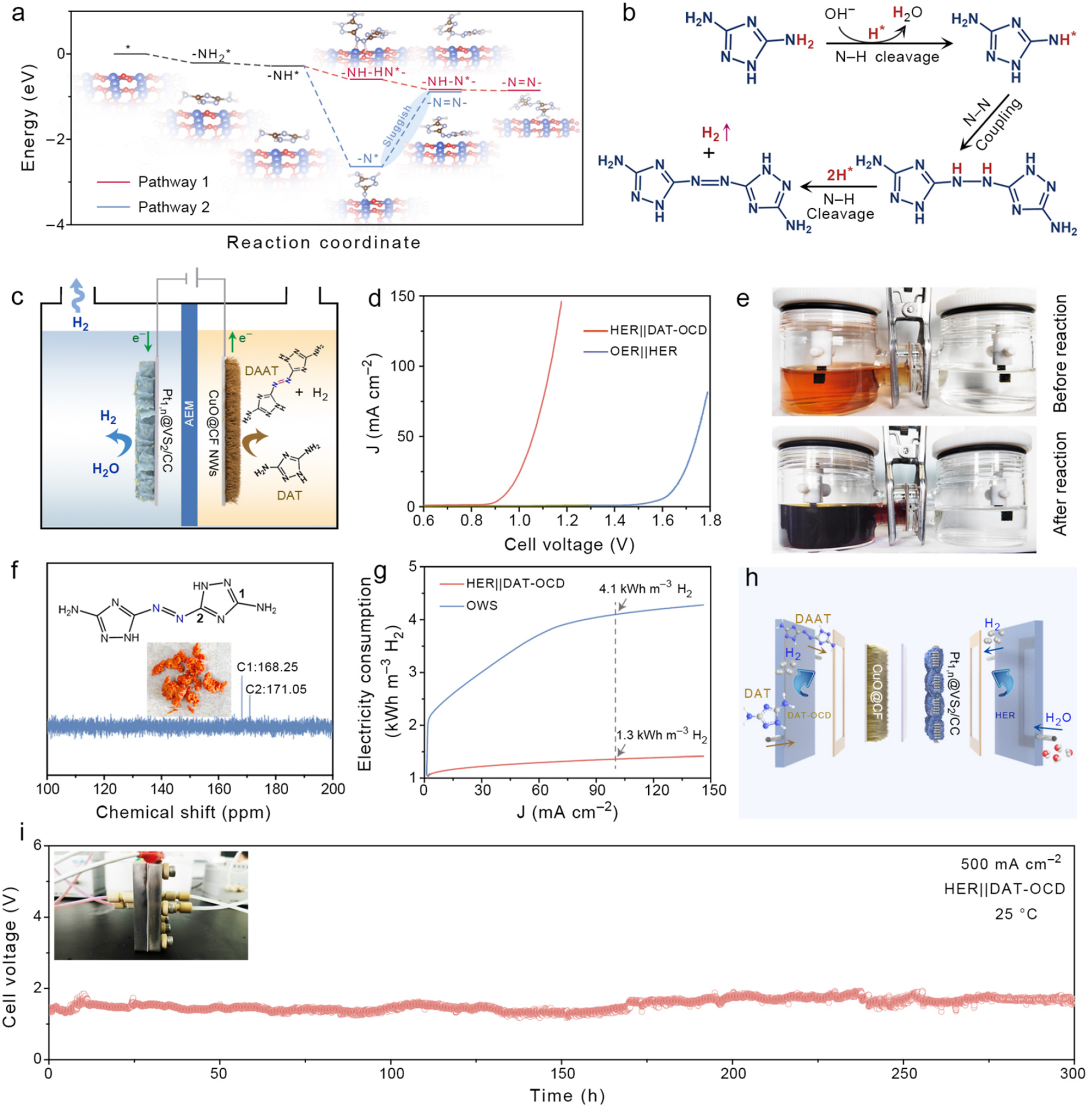
(a) Relative energy of a possible DAT-OCD pathway. (b) Proposed DAT-OCD mechanism on CuO@CF NWs. (c) Schematic illustration of the H-type cell for HER||DAT-OCD. (d) LSV curves of the traditional OWS and coupling system of HER||DAT-OCD. (e) Optical image of the coupling system before and after the long-term stability test. (f) ^13^C NMR spectrum of DAAT products. (g) Consumed electricity analysis of the OWS and HER||DAT-OCD. (h) Schematic illustration of an AEM electrolyzer. (i) Long-term stability test of the HER||DAT-OCD AEM electrolyzer at 500 mA cm^–2^.

DFT calculations were implemented to further verify the possible direct electrooxidation pathway for DAT-OCD (Figure 5a). Compared with the adsorption energy (*E*_ads_) of the vertically aligned DAT molecule on CuO (*E*_ads_ = 0.3 eV) (Figure S23), the horizontally aligned DAT molecule exhibits a lower *E*_ads_ (−0.21 eV), which is used as a model structure for the following calculations. All of the dehydrogenation and −NH–NH– coupling steps are exothermic processes in pathway 1, while the sluggish step is observed on −N* and −N* coupling to form −N=N– final products in pathway 2, indicating that pathway 1 dominates the DAT-OCD process. In addition, the free energies of amino dehydrogenation of DAT molecules on Cu and Cu(OH)_2_ were assessed by DFT calculations. As shown in Figure S24, the free energies of amino dehydrogenation on Cu and Cu(OH)_2_ were 2.03 and 2.25 eV, respectively, while the amino dehydrogenation was more favorable on CuO (−0.28 eV, Figure 5a), suggesting a weak amino dehydrogenation capacity on Cu and Cu(OH)_2_ catalysts. It is noted that the O–H in Cu(OH)_2_ tends to dissociate and the produced H* bonds with dehydrogenated animo to form a DAT molecule, indicating that the dehydrogenation of DAT was thermodynamically unfavorable on these catalysts. This may be the reason for the enhanced DAT-OCD electrocatalytic activity on CuO@CF, which is consistent with the electrochemical results and the in situ ATR-FTIR analysis. Based on the above discussion, we propose that the main mechanistic pathway of dehydrogenation of the amino group driven by OH* in DAT yields −NH* intermediates and water molecules. Then, the −NH* reacts with another −NH* to achieve N–N oxidative coupling, leading to −NH–NH– intermediates. Finally, dehydrogenation proceeds further to yield the DAAT products, while the produced H* intermediates combine to form H_2_ via the Tafel pathway (Figure 5b).

### Water Electrolysis Device Performance

Encouraged by the superior electrocatalytic performance of HER and DAT-OCD, a two-electrode cell employing Pt_1,n_@VS_2_/CC and CuO@CF NWs as the cathode and anode electrodes was constructed, respectively (Figure 5c). The HER||DAT-OCD delivers ultralow cell voltages of 0.94 and 1.05 V to reach 10 and 50 mA cm^–2^, respectively, remarkably lower than that of HER||OER (overall water splitting, OWS), where high cell voltages of 1.67 and 1.74 V are required at 10 and 50 mA cm^–2^, respectively (Figure 5d). The excellent activity of HER||DAT-OCD is attributed to the low-potential N–N oxidative coupling reaction, which in turn increases the cathodic hydrogen production energy efficiency.

The stability after the third LSV test was determined, and the results are shown in Figure S25. CuO@CF exhibits no apparent morphology changes. The long-term stability of the H-type cell for HER||DAT-OCD is then determined at a current density of 10 mA cm^–2^ (Figure S26). The coupled system steadily operated for at least 300 h with negligible activity attenuation. The sudden voltage “drop” during the test is due to the supplementation of the DAT substrate, indicating the DAT-OCD is crucial for low-energy H_2_ production.

After the long-term stability reaction, the color of the anodic electrolyte changes from orange red to crimson (Figure 5e). The obtained anodic electrolyte is then treated by acidification, hot filter, and vacuum drying (as detailed in the Experimental Section in the SI). The target orange-colored DAAT products were collected and characterized by NMR spectroscopy (Figure 5f). The two kinds of carbon environments located at the chemical shift of δ = 168.25 and 171.05 ppm are assigned to C–NH_2_ and C–N=N species, respectively.^34^ No other compounds, including dehydrogenation of the para position of amino to yield oligomers, are detected by ^13^C NMR spectroscopy, indicating the successful synthesis of DAAT compounds (Figure S27). Benefiting from the bipolar hydrogen production on both anodic N–N oxidative dehydrogenation and cathodic hydrogen evolution, the electricity consumption of the coupling system only required 1.3 kWh m^–3^ H_2_ at a current density of 100 mA cm^–2^, which is one-third the electricity of conventional OWS (4.1 kWh m^–3^ H_2_) to reach the same current density, saving ∼68% electricity input (Figure 5g).

Encouraged by the excellent performance of the bipolar hydrogen production system, we further assembled an anion-exchange membrane (AEM) electrolyzer to evaluate the industrial potential of our bipolar hydrogen production DAT-OCD||HER system using CuO@CF NWs and Pt_1,n_@VS_2_/CC as the anode and cathode, respectively (Figure 5h). The long-term stability of the AEM electrolyzer is tested at a current density of 500 mA cm^–2^, demonstrating the excellent durability over 300 h with negligible activity decay (Figure 5i). The Faradaic efficiency (FE) of anodic DAT-OCD was calculated at various current densities (Figure S28). The FE was maintained at a high level of >90% over a wide current density range. The DAT-OCD can also deliver a high FE of 90.8% at a large current density of 500 mA cm^–2^. The commendable performance of the DAT-OCD||HER system suggests the potential of the industrialized alkaline water electrolyzer.

## Conclusions

A hybrid low-potential electrolyzer system was successfully developed, combining an N–N oxidatively coupled dehydrogenation reaction of the amino group in aminoazole substrates and the usual HER for anodic and cathodic hydrogen productions, respectively. An ultralow potential of 0.87 V vs RHE is obtained for DAT-OCD at 10 mA cm^–2^. In situ DEMS, isotope labeling tests, in situ ATR-FTIR spectroscopy, and DFT calculations confirm that hydrogen production during DAT-OCD and the H atoms in H_2_ are all from the amino group in the DAT substrates. The N–H bond cleavage and −NH–NH– bridging followed by further dehydrogenation to form −N=N– dominate the reaction. Meanwhile, the H atoms from N–H bond cleavage are recombined through the Tafel pathway to form H_2_. The cathodic alkaline HER is driven by highly efficient Pt_1,n_@VS_2_/CC electrocatalysts with a low η_10_ of 40.3 mV and a superior stability of 100 h. DFT calculations demonstrate the cascade catalysis of alkaline HER and that the Pt SAs sites of Pt_1,n_ promote the Volmer step, which causes H* to spill over to the S sites via the Heyrovsky pathway for fast hydrogen production.

A coupled H-type electrolysis system was assembled, and ultralow cell voltages of 0.94 and 1.05 V were required to reach 10 and 50 mA cm^–2^, respectively. Consequently, this coupled system only requires one-third of the electricity of the conventional OWS. Remarkably, a hybrid flow cell of the AEM device with DAT-OCD||HER shows robust stability at 500 mA cm^–2^ for 300 h, revealing great promise of the hybrid system for large-scale applications. Given the low-potential N–N oxidative dehydrogenation for hydrogen production at the anode, we anticipate that this bipolar hydrogen production system will be applicable to many amino group-based dehydrogenation processes for low-energy hydrogen production, as well as value-added azo chemicals from the anode.

## Supporting Information Available

Additional experimental details, materials, and methods; SEM and TEM images of VS_2_@CC; EPR, contact angle tests, XPS of Pt_1,n_@VS_2_/CC and VS_2_/CC; EDTA and KSCN poison test of HER; structural models of VS_2_-supported Pt_1_, Pt_n_, and Pt_1,n_; structural models of H* adsorption and water dissociation sites on Pt_1_@VS_2_, Pt_n_@VS_2_, and Pt_1,n_@VS_2_; in situ SERS of HER at various potentials; in situ ATR-FTIR spectra of DAT-OCD and OER; SEM image of Cu(OH)_2_@CF; XRD pattern of CuO@CF; in situ DEMS of OER and DAT-OCD; ^1^H and ^13^C NMR spectra of DAT and DAAT; CP test of the H-type coupled system; FE of DAT-OCD; table of Pt *L*_3_-edge EXAFS fitting parameters for all samples; table of comparison of applied potential for DAT-OCD with references (PDF)
Anodic H_2_ production from DAT-OCD (MP4)
