# Supplementary material for: Electrochemical N–N Oxidatively Coupled Dehydrogenation of 3,5-Diamino-1H-1,2,4-triazole for Value-Added Chemicals and Bipolar Hydrogen Production
Source: J Am Chem Soc. 2025 Mar 8;147(11):9505–18. doi: 10.1021/jacs.4c17225 (PMC11926877; doi:10.1021/jacs.4c17225)
Supplement: Supplementary file 1 — ja4c17225_si_002.pdf [file ja4c17225_si_002.pdf]

Electrochemical N–N Oxidatively Coupled Dehydrogenation of 3,5-Diamino-1*H*-1,2,4-triazole for Value-Added Chemicals and Bipolar Hydrogen Production

Jiachen Li, Yang Li, Yuqiang Ma, Zihang Zhao, Huarong Peng, Tao Zhou, Ming Xu, Daidi Fan, Haixia Ma,* Jieshan Qiu*, Zhengxiao Guo**

Methods

Chemicals

Carbon cloth was purchased from Shanghai Sanmusk Industrial Co., Ltd. Copper foam was obtained from Suzhou ZhengTairong Electronic Materials Co., LTD. Sodium orthovanadate ($Na_3VO_4·12H_2O$, AR, ≥99%), thioacetamide (C_2H_5NS, AR, ≥98.0%), chloroplatinic acid hydrate ($H_2PtCl_6·xH_2O$), ammonium persulfate ($(NH_4)_2S_2O_8$, AR, ≥98%), 3,5-Diamino-1,2,4-triazole ($C_2H_5N_5$, AR, ≥98%) were purchased from Shanghai Aladdin Biochemical Technology Co., LTD. All the reagents were used without further purification.

Preparation of cathodic $Pt_{1,n}$@VS_2/CC catalysts

The $Pt_{1,n}$@VS_2/CC was prepared by a two-step method. Before the reaction, the CC substrates were treated with acetone to remove the oil stain. Then, the pre-treated CC (1 × 3 cm^2) was immersed in the 30 mL aqueous solution containing $NH_4VO_3·12H_2O$ (2 mmol), C_2H_5NS (20 mmol), and ammonium hydroxide (2 mL). The obtain uniform solution was transferred to a Teflon-stainless steel reactor. The hydrothermal reaction was carried out at 180 ℃ for 20 h. After cooling down, the green-colored VS_2 NSs supported on CC was obtained (VS_2/CC NSs). The $Pt_{1,n}$ loaded on the VS_2/CC NSs was prepared by a simple cyclic voltammetry (CV) electrodeposition method. Typically, the electrochemical depositions were carried out from –0.6 to 0.3 V *vs.* RHE for cathodic deposition with a sweeping rate of 50 mV s^{-1} for 200 cycles until the CV curves tend to be stable. The loading of Pt in $VS_2/Pt_{1,n}$ was well controlled by adjusting the volume of $H_2Cl_6Pt·xH_2O$ (10 mg mL^{-1}) in 1.0 M KOH solution, in which the 0., 0.5, 1.0, 2.0 and 3.0 mL were considered and abbreviated as Pt-0.1, Pt-0.5, Pt-1.0, Pt-2.0 and Pt-3.0, respectively. The electrodeposition was conducted in a three-electrode system that the VS_2/CC NSs, Hg/HgO, and a high-purity graphite rod was used as the working electrode, the reference electrode, and the counter electrode, respectively. After the deposition, the obtained $Pt_{1,n}$@VS_2/CC was washed with deionized water for several times and then directly used as self-supporting cathodic electrodes for subsequent

alkaline HER performance tests

Preparation of anodic CuO@CF NWs catalysts

The CuO@CF NWs were prepared by facile thermal chemical process. Before the reaction, the CF (1×1 cm^2) was treated by 1.0 M HCl and ultrasonic treatment for 30 min to remove oxides. The CF was then washed with deionized water for several times. The treated CF was subsequently immersed in the aqueous solution involving 3.0 M NaOH and 0.6 M $(NH_4)_2S_2O_8$ and maintained for 1 h. The obtained blue-colored $Cu(OH)_2$@CF NWs were washed with distilled water to remove the remaining reactants of the $Cu(OH)_2$@CF surface. The CuO@CF NWs was obtained by calcining $Cu(OH)_2$@CF in muffle furnace at 300 ℃ for 4 h with the heating rate of 5 ℃ min^{-1}. The obtained wine red-colored CuO@CF NWs were directly used as self-supporting anodic electrodes for DAT-OCD performance.

Material characterizations

SEM was carried out on a Hitachi, SU8010. TEM and HAADF-STEM elemental mappings were performed on a FEI Talos F200X. $Pt_{1,n}$ on VS_2 was verified by a spherical aberration-corrected STEM images (FEI Themis Z). XRD was performed on an X-ray diffractometer (Bruker D8 Advance) with Cu–*K*α radiation. XPS was carried out on a Thermo Scientific Nexsa spectroscopy. Raman spectra were taken on a Thermo Fisher DXR2 Xi with a green laser of 532 cm^{-1}. The products of DAT-OCD and deuterated DAT substrates were detected by ^{13}C and ^{1}H NMR on a Bruker Advance 400 in D_2O at 25 °C. The content of $Pt_{1,n}$ in $VS_2/Pt_{1,n}$ was recorded by an ICP-MS (Agilent 7800). XAS was recorded at the beamline 1W1B of the Beijing Synchrotron Radiation Facility (BSRF, operated at typical energy of the storage ring of 2.5 GeV). Calibration with standard Pt foil and PtO_2 and the samples were tested using fluorescence mode. *In situ* surface-enhanced Raman spectroscopy (SERS) tests were performed at a Horiba LabRAM Odyssey instrument at a laser wavelength of 532 nm.

***In situ* differential electrochemical mass spectrometry (DEMS)**

The gaseous products of anodic DAT-OCD were detected in a *in situ* DEMS (Shanghai Linglu Instrument Equipment). The electrolyte and vacuum system were separated by a Teflon film to adequate filtration the gaseous products and minimize the mixture of reagents. The vacuum degree is maintained below 1×10^{-7} Pa. The trace amount of gas product was then detected by *in situ* DEMS.

***In situ* attenuated total reflection Fourier-transform infrared (ATR-FTIR)**

The DAT-OCD mechanism was investigated by *in situ* FTIR on a Nicolet IS50 spectroscopy with a mercury cadmium telluride (MCT) detector equipped with Pike Technologies VeeMAXIII and Jackfish J1 accessory. The Au film was deposited on the Si crystal by vacuum spraying as the conductive substrates for CuO NWs catalysts loading. The CuO NWs powder catalysts were prepared by ultrasonically treated of CuO@CF NWs (1×1 cm^2) in aqueous solution containing ethanol (0.5 mL), distilled water (0.5 mL), and Nafion solution (40 μL, Dopant D520, 5%). The prepared ink was then doped on the surface of Au foil and air dried.

Electrochemical characterizations

All the electrochemical tests are performed at a electrochemical workstation (CHI 760e, Chenhua, Shanghai). The cathodic alkaline HER tests were performed under three-electrode system. The prepared $Pt_{1,n}$@VS_2/CC, high-purity graphite rod, and Hg/HgO were used as working electrode, counter electrode, and reference electrodes, respectively. 1.0 M KOH aqueous solution was the electrolyte. LSV curves were recorded over the potential ranges of –0.6 V to 0.3 V *vs.* RHE with a scan rate of 2 mV s^{-1}. Stability of the catalysts were performed by chronopotentiometry method. The anodic DAT-OCD was carried out under three-electrode system. CuO@CF NWs, Hg/HgO, and Pt wire were used as working electrode, reference electrode, and counter electrode, respectively. The electrolyte contains 1.0 M KOH aqueous solution with various concentrations (0.1, 0.2, 0.3, 0.4 M) of DAT substrates. The LSV of DAT-OCD were recorded over the potential ranges of 0.6~1.6 V *vs.* RHE. All the potentials of the

three-electrode system were calibrated with respect to the RHE by the Eq:

$$E_{(RHE)} = E_{(Hg/HgO)} + 0.098\ \text{V} + 0.0591 \times pH \quad (1)$$

The H-type cell of HER and DAT-OCD were assembled on a two-electrode system with CuO@CF NWs and $Pt_{1,n}$@VS_2/CC as anode and cathode, respectively. The 1.0 M KOH and 1.0 M KOH+0.2 M DAT were respectively used as cathodic and anodic electrolytes, which were divided by a piece of anion-exchange membrane (Fumasep FAA-3-50). The AEM flow cell device was assembled with an anode and cathode (1.0 cm^2), an anion exchange membrane (Fuma FAA-PK-130). The prepared $Pt_{1,n}$@VS_2/CC and CuO@CF NWs were directly used as gas diffusion layers for cathode and anode to construct $Pt_{1,n}$@VS_2/CC||CuO@CF NWs AEM electrolyzer without additional process (pressing or heating). The flow cell performance was studied in a 1.0 M KOH and 1.0 M KOH+0.2 M DAT for catholyte and anolyte respectively at room temperature using CHI 760e (Chenhua, Shanghai) equipped with a current amplifier. The method of DAAT product separation is based on solubility difference. The pH was adjusted to neutral with sulfuric acid, stirred at 100 °C for 30 min, and treated with hot filter. Because DAAT is insoluble in methanol, chloroform, toluene, DMSO, DMF, and is only slightly soluble in water at high temperature, impurities are removed by heating filtration, and drying the filtered products in a vacuum oven for 12 h to obtain a solid of orange color. The stability test was carried out by chronopotentiometry at a current density of 500 mA cm^{-2} for 500 h. The electrolytes were treated with irregular renewal because of the fast consumption of DAT substrates and water during the long-term stability evaluation.

HER intrinsic activity evaluation

The conversion efficiency of $Pt_{1,n}$@VS_2 was evaluated by the TOF values (s^{-1}), which can be obtained according to the Eq:

$$\text{TOF} = \frac{Total\ number\ of\ H_2\ turnover/geometric\ area\ (cm^2)}{Total\ number\ of\ active\ sites/geometric\ area\ (cm^2)} \quad (2)$$

The total number of H_2 turnovers were calculated by the Eq:

$$\text{Total } H_2 \text{ turnovers} =$$

$$[J\ (\text{mA cm}^{-2})]\left[\frac{1 (C\ s^{-1})}{10^3\ (mA)}\right]\left[\frac{1\ (mol\ e^{-})}{96485\ (C)}\right]\left[\frac{1\ mol\ H_2}{2\ mol\ e^{-}}\right]\left[\frac{6.02\times10^{23}\ molecules\ H_2}{1\ mol\ H_2}\right]$$

The number of active sites was calculated by the total mass of the Pt quantified by the ICP-MS. The current density (J) from LSV can be converted to TOF according to the Eq:

$$\text{TOF} = \frac{3.12\times10^{15}}{Number\ of\ active\ sites} \times |J|\ (\text{s}^{-1}) \qquad (3)$$

The atomic utilization of Pt in $Pt_{1,n}@VS_2$ was calculated by the mass activity according to the Eq:

$$J_{mass}^{Pt} = \frac{J^{Pt_{1,n}@VS_2/CC} - J^{VS_2@CC}\ (mA\ cm^{-2})}{mass_{Pt}\ (mg\ cm^{-2})} \qquad (4)$$

Electricity consumption calculation

The electricity consumption (W, kWh per m^3 of H_2) of the bipolar hydrogen production for DAT-OCD||HER coupled system was calculated by the Eq:

$$W = \frac{n\times F\times U\times 1000}{3600\times V_m} \qquad (5)$$

Where n is the transferred electron to produce each H_2 molecule. Note that n=1 in our bipolar hydrogen production system while n=2 for conventional OWS. U is the applied cell voltage and V_m (22.4 mol L^{-1}) is the molar volume of gas under standard conditions. F is the Faradic constant (96485 C mol^{-1}).

Theoretical calculations

DFT calculations were performed by Vienna Ab initio Simulation Package (VSAP).[74] Generalized gradient approximation (GGA) and Perdew-BurkeErnzerhof (PBE) were used to calculate the exchange-correlation energy. A cutoff energy of 500 eV was employed in all simulations. The polarizable implicit solvent models with the dielectric constant was set to 78.4 and was included the VASPsol.[75] For cathodic catalysts model, one Pt atom, Pt_{13} NCs, and coexistence of Pt atom and Pt_{13} NCs were constructed on the sulfur vacancy of VS_2 (002) plane based on the structural characterizations of

$VS_2/Pt_{1,n}$. For anodic catalysts model, CuO (111) and $Cu(OH)_2$ (002) planes were selected for DAT adsorption and DAT-OCD pathway investigations. Structural optimization was carried out with full relaxation until the energy and forces were reduced to less than 10^{-5} eV and 0.02 eV Å^{-1}, respectively. A vacuum spacing of 15 Å was set for the slab to avoid the interaction between the periodic images. The water dissociation energy barrier was evaluated by transitional state searching using the Nudged Elastic Band method. The free energy of ΔG was calculated based on the Eq:

$$\Delta G = \Delta E_{\text{ads}} + \Delta E_{ZPE} - T\Delta S \tag{6}$$

where ΔE_{ads} is the adsorption energy, ΔZPE and ΔS denote the zero-point energy and entropy change. T is temperature ($T = 298.15$ K). The ΔE_{ads} are calculated by the Eq:

$$\Delta E_{\text{ads}} = E_{(*+\text{ad})} - E_{(*)} - E_{(\text{ad})} \tag{7}$$

where $E_{(*+\text{ad})}$, $E_{(*)}$, and $E_{(\text{ad})}$ represent the total energy of adsorbate adsorbed on the surface, the energy of clean surface, and the adsorbate molecules, respectively.

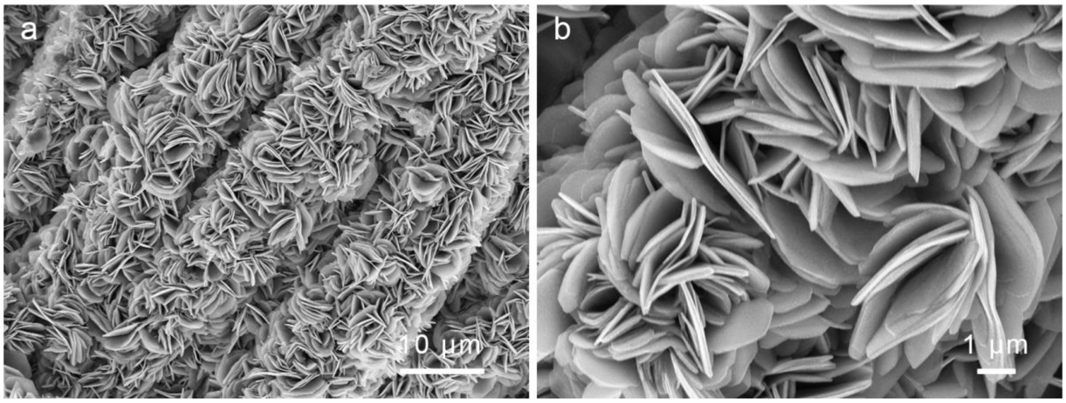

Figure S1. (a) Low- and (b) high-resolution SEM images of VS_2@CC NSs.

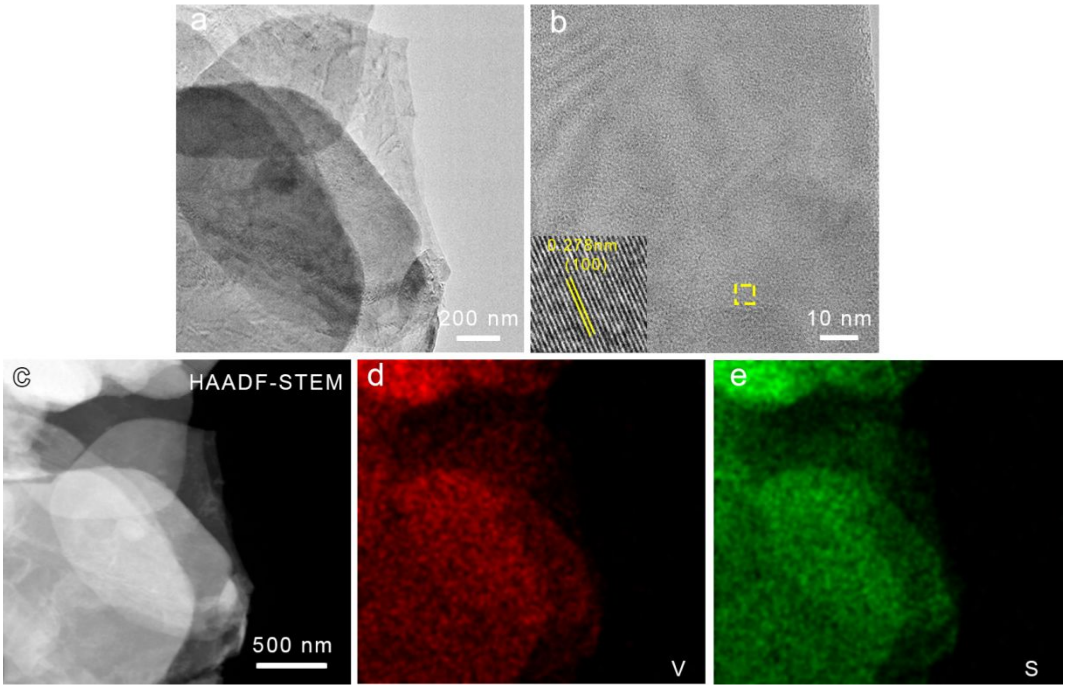

Figure S2. (a, b) Low and high-resolution TEM images of VS_2 NSs. (c~e) HAADF-STEM and corresponding elemental mappings of V and S in VS_2.

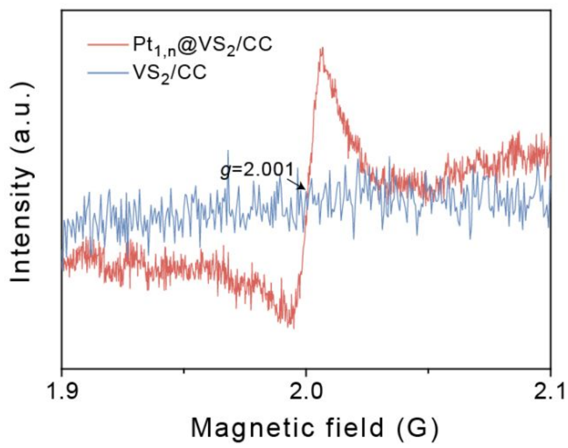

Figure S3. EPR spectra of VS_2@CC and $Pt_{1,n}$@VS_2/CC.

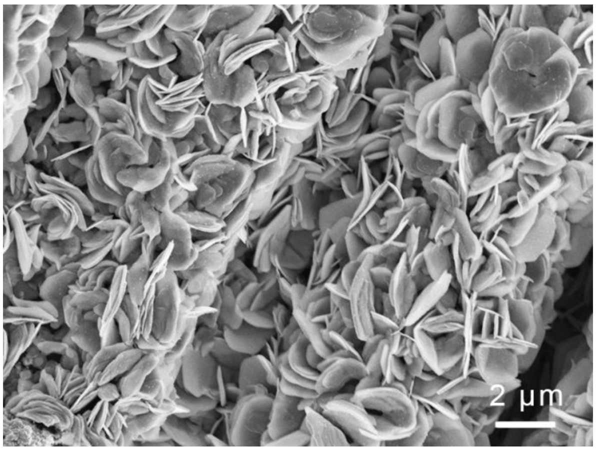

Figure S4. Low-resolution SEM image of $Pt_{1,n}$@VS_2/CC.

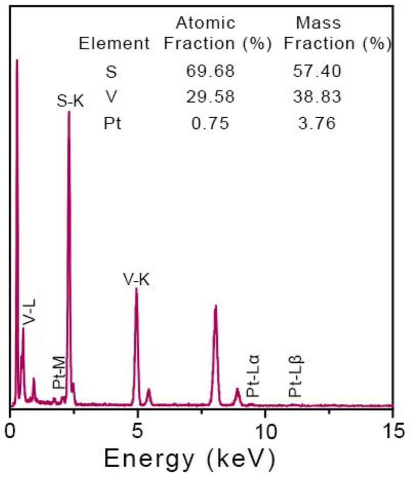

Figure S5. EDS spectrum of $Pt_{1,n}@VS_2$.

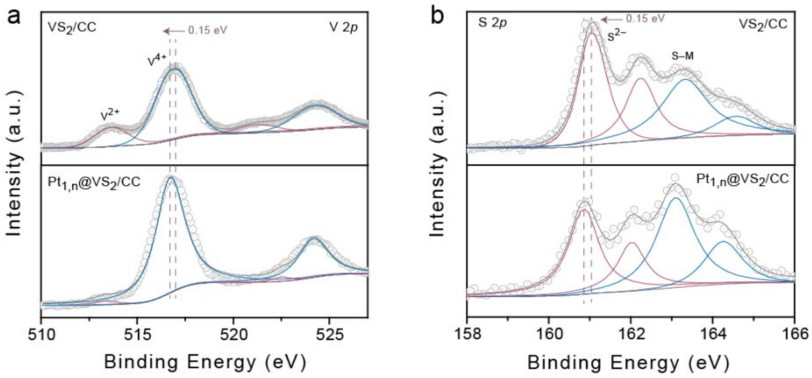

Figure S6. XPS spectra of (a) V 2p and (b) S 2p signals for VS_2@CC and $Pt_{1,n}$@VS_2/CC.

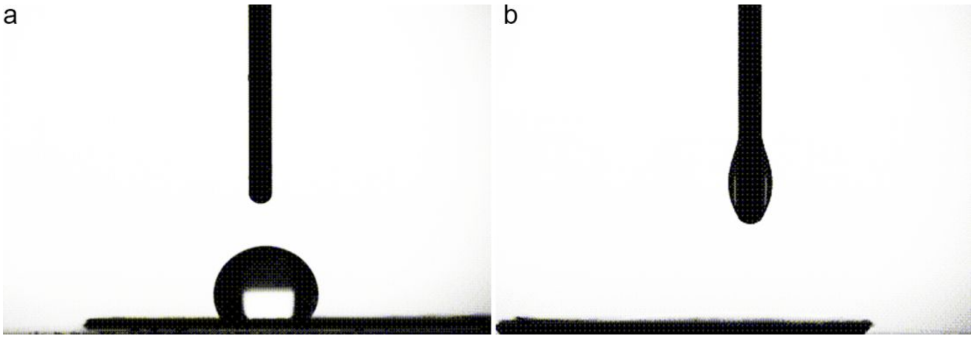

Figure S7. Contact angle of water droplets sitting on the surface of the (a) CC substrate, (b) $Pt_{1,n}$@VS_2/CC.

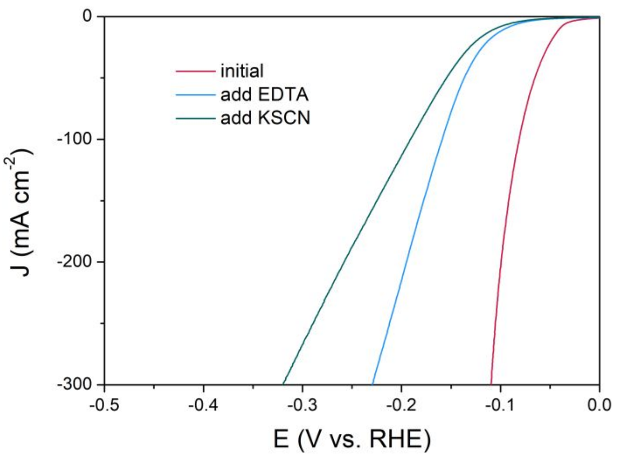

Figure S8. LSV curves of $Pt_{1,n}$@VS_2/CC with the addition of 5 mM EDTA and KSCN respectively in 1.0 M KOH aqueous solution.

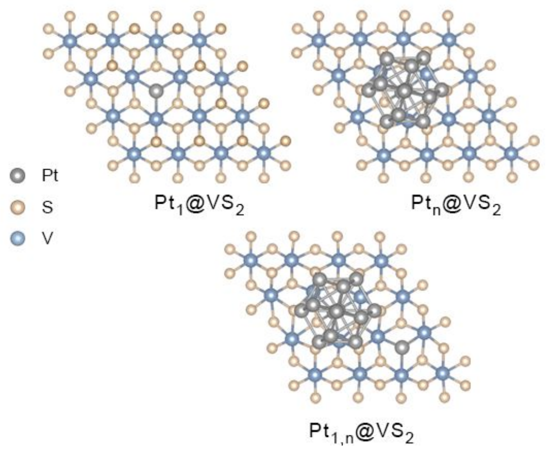

Figure S9. Structural models of VS_2 supported Pt_1, Pt_n, and $Pt_{1,n}$, respectively.

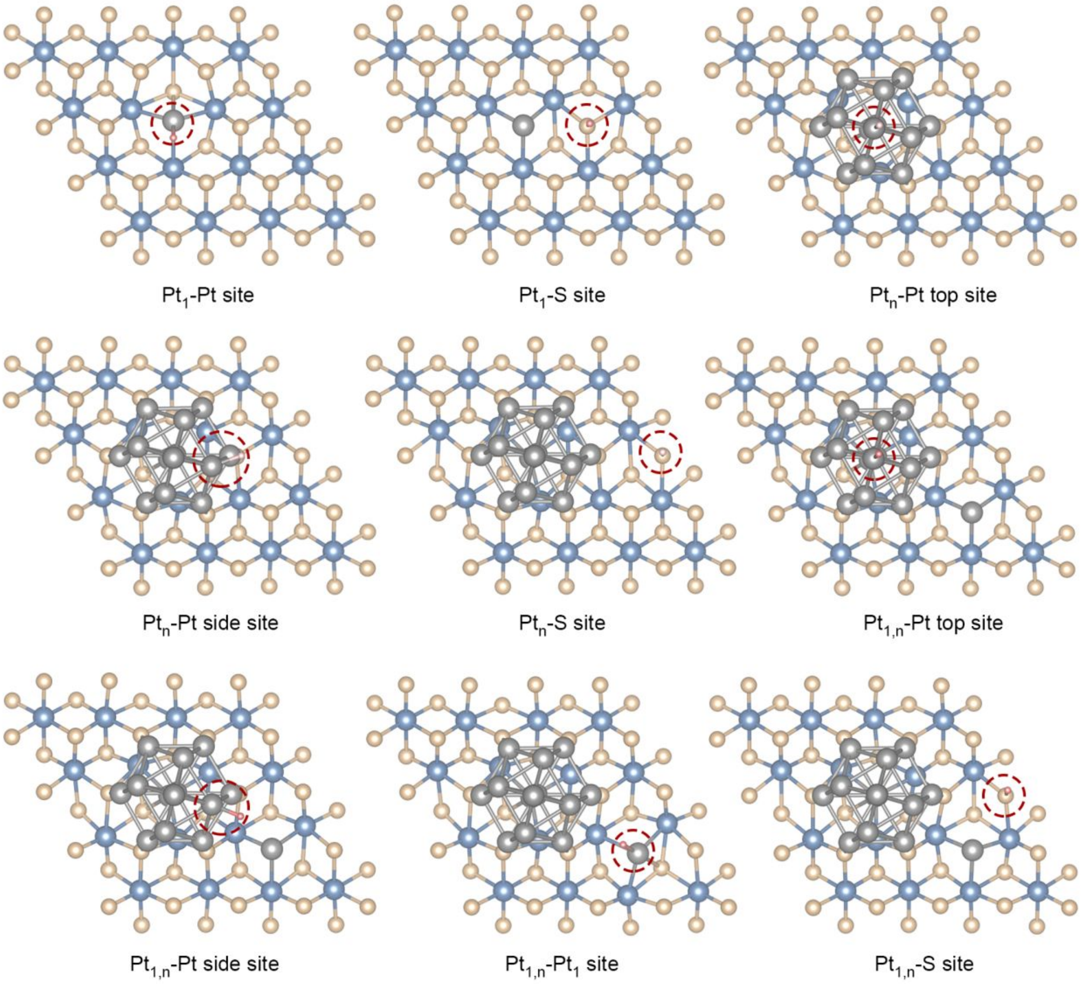

Figure S10. Structures of different H*-adsorption sites on Pt_1@VS_2, Pt_n@VS_2, and $Pt_{1,n}$@VS_2.

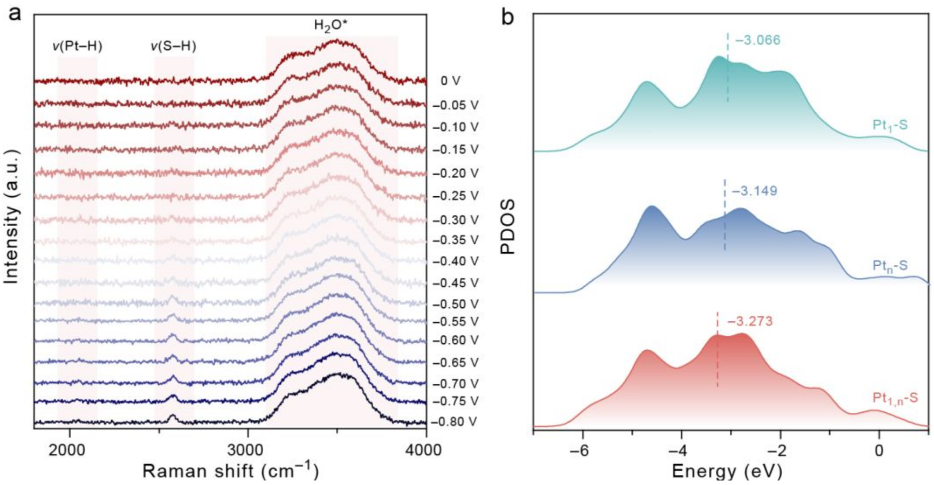

Figure S11. *In-situ* Raman spectra of HER on $Pt_{1,n}$@VS_2 in 1.0 M KOH over the potential ranges of 0 ~ –0.8 V vs. RHE. (b) The *p* orbitals density of state of S atoms on the surfaces of $Pt_{1,n}$@VS_2, Pt_1@VS_2, and Pt_n@VS_2.

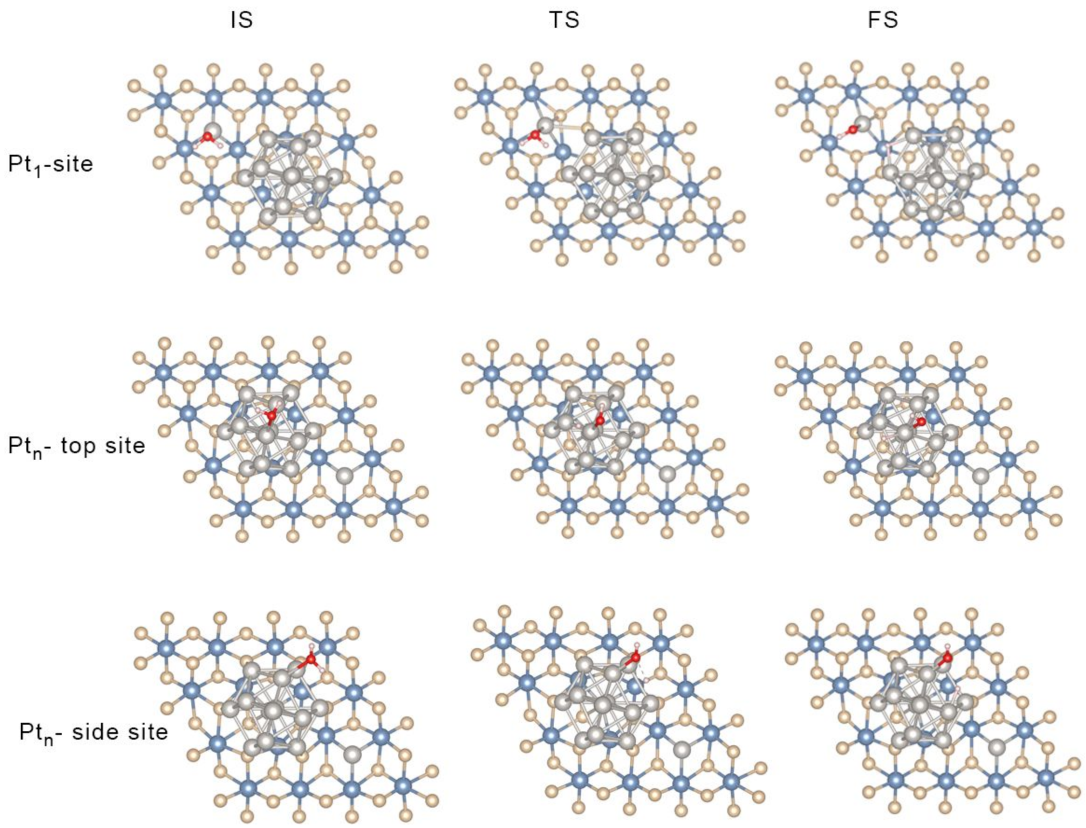

Figure S12. Structural models of initial state, transition state, and final state of Volmer pathway on Pt SA site, Pt NC top site, and Pt NC side site.

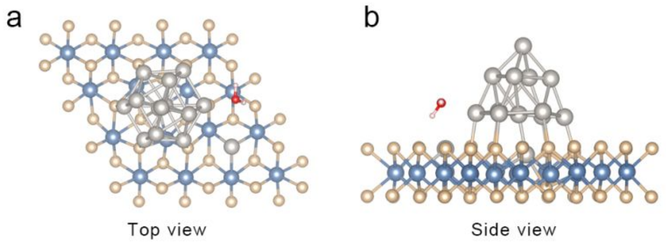

Figure S13. Structural models of H_2O molecule adsorbed on S sites of $Pt_{1,n}@VS_2$ (a) top view and (b) side view

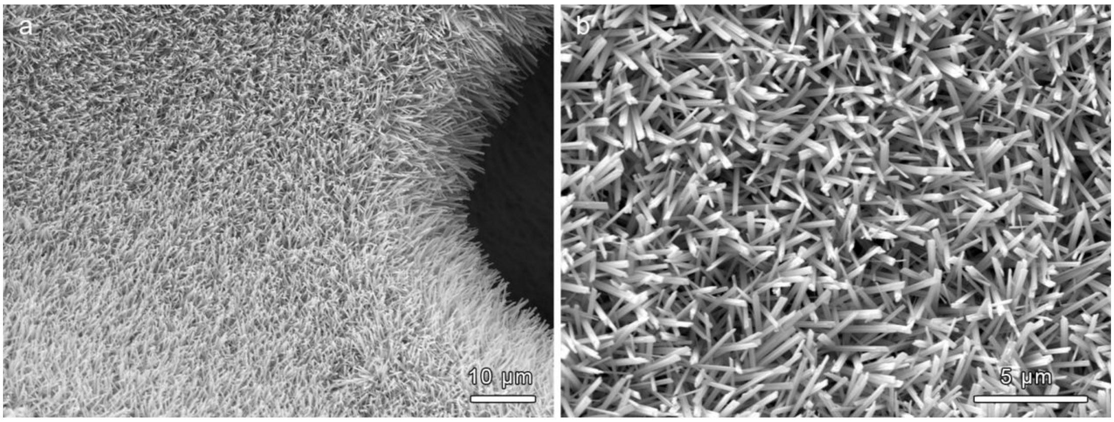

Figure S14. (a, b) Low and high-resolution SEM images of $Cu(OH)_2$@CF NWs.

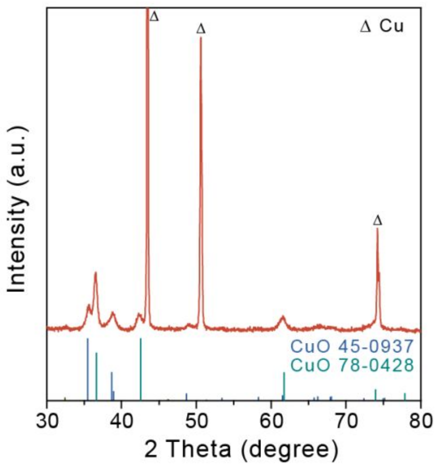

Figure S15. XRD pattern of CuO@CF.

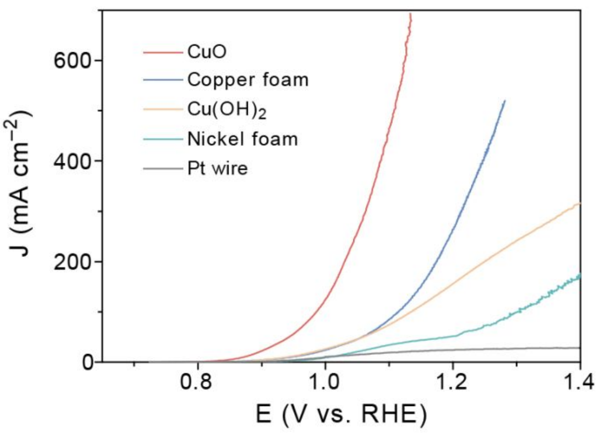

Figure S16. LSV curves of DAT-OCD on different electrocatalysts.

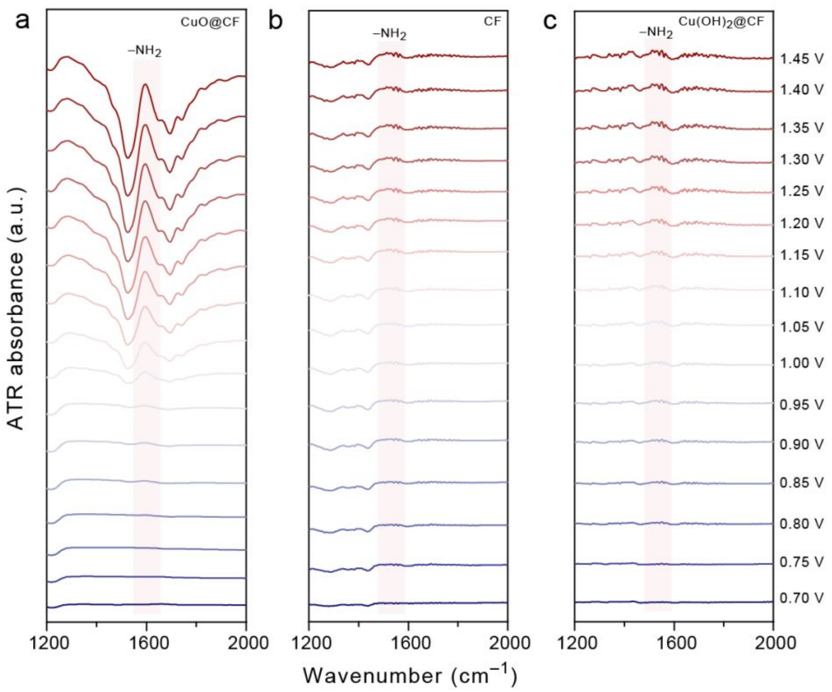

Figure S17. *In situ* ATR-FTIR of DAT-OCD at the wavenumber range of 1200~2000 cm^{-1} for (a) CuO@CF, (b) CF, and (c) $Cu(OH)_2$@CF.

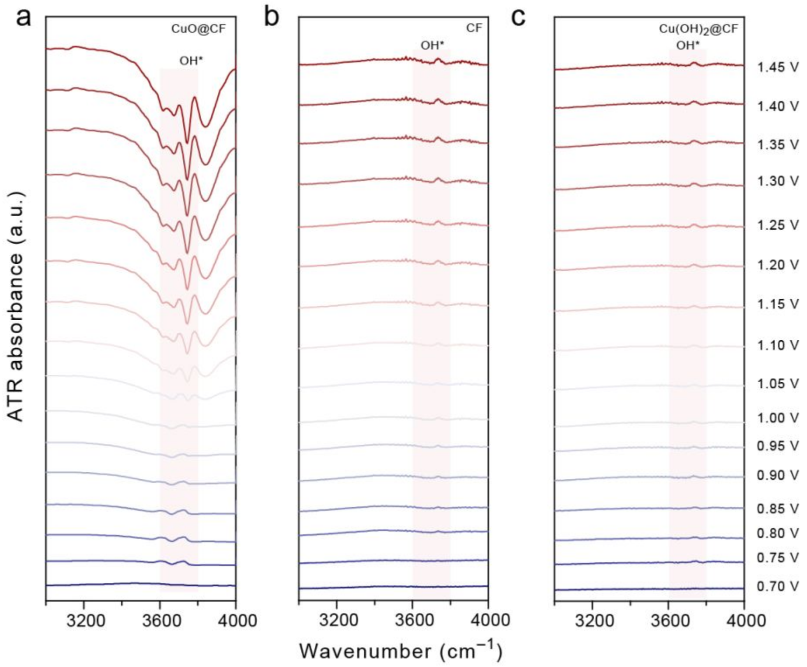

Figure S18. *In situ* ATR-FTIR of DAT-OCD at the wavenumber range of 3000~4000 cm^{-1} for (a) CuO@CF, (b) CF, and (c) $Cu(OH)_2$@CF.

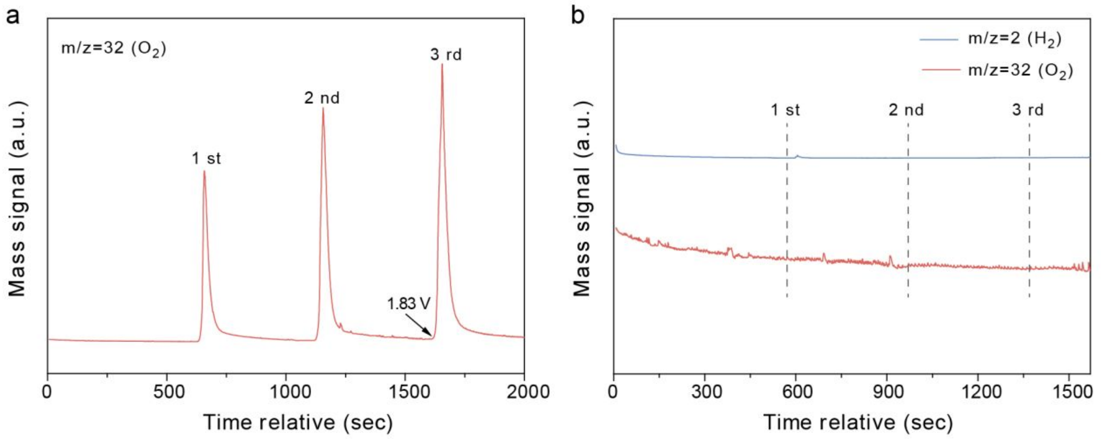

Figure S19. In-situ DEMS spectra of OER on CuO@CF NWs over the potential range of (a) 0.7–2.2 V vs. RHE. (b) 0.7–1.4 V vs. RHE.

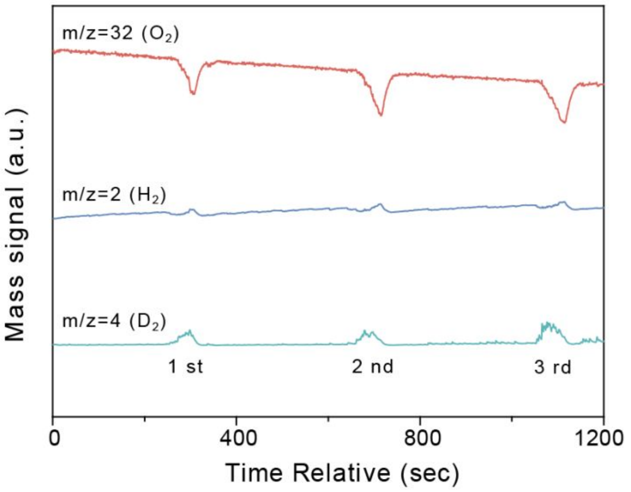

Figure S20. In-situ DEMS spectra of deuterated DAT-OCD on CuO@CF NWs over the LSV potential range of 0.7–1.7 V vs. RHE in 0.2 M DAT+1.0 M KOH.

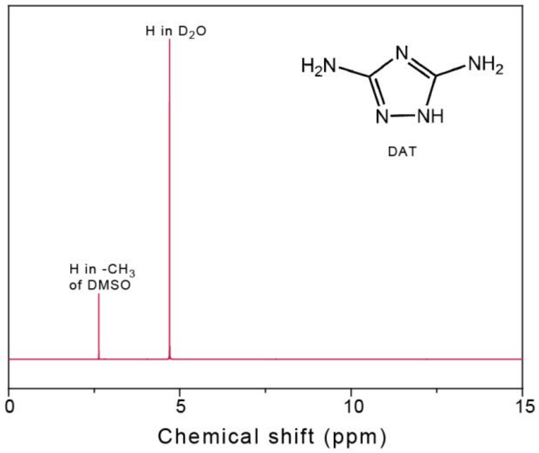

Figure S21. ^{1}H NMR spectrum of DAT substrate material in D_2O and further treated with DMSO.

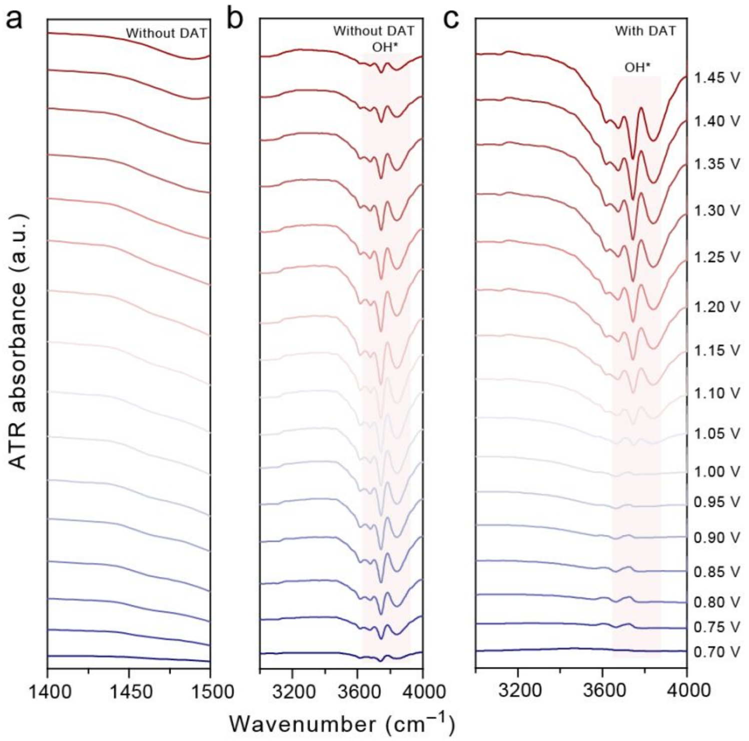

Figure S22. *In situ* ATR-FTIR spectra of DAT-OCD and OER over selected wavenumber ranges.

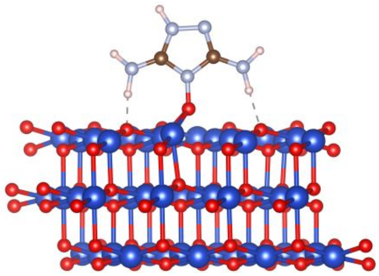

Figure S23. Structural model of DAT vertically adsorbed on the CuO surface.

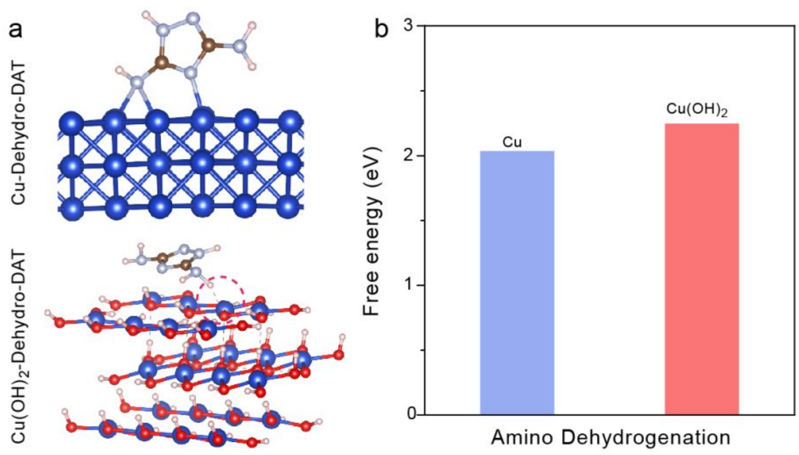

Figure S24. (a) Structural models of dehydrogenated DAT adsorbed on Cu and $Cu(OH)_2$. (b) Calculated free energy of amino dehydrogenation of DAT molecule on Cu and $Cu(OH)_2$.

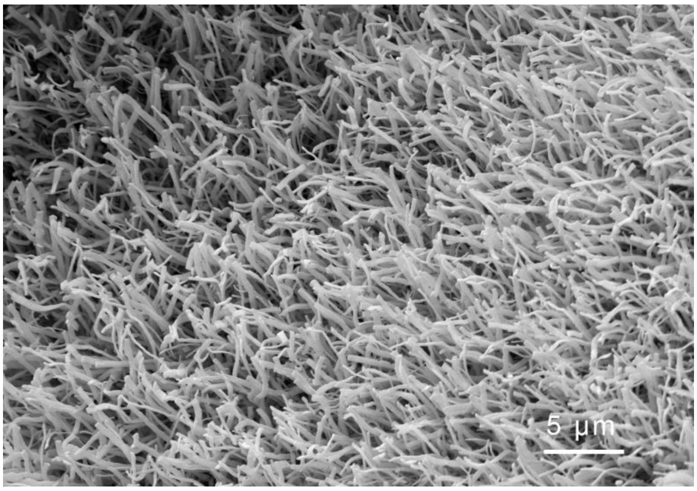

Figure S25. SEM images of CuO@CF after 3rd LSV tests of DAT-OCD||HER.

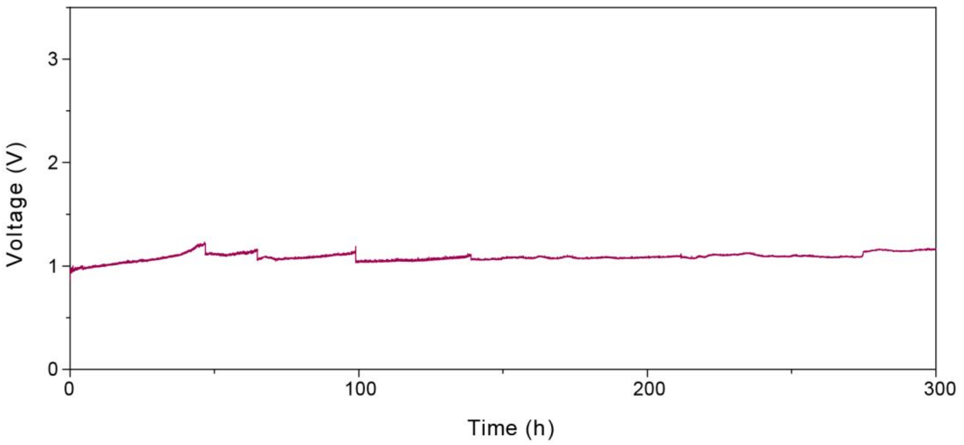

Figure S26. Long-term stability test of H-type cell for DAT-OCD||HER at the current density of 10 mA cm^{-2}.

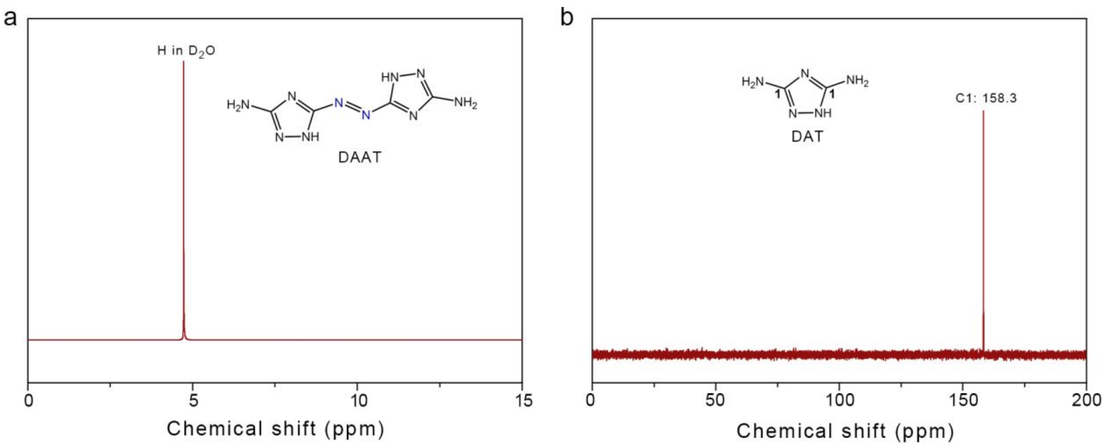

Figure S27. (a) ^{1}H NMR spectrum of DAAT product. (b) ^{13}C NMR spectrum of DAT substrate.

As the reactive hydrogen in the amino group and the triazole unit rapidly undergoes H–D exchange with the solvent (deuterium oxide), the hydrogen signal was not detected in the ^{1}H NMR spectra of DAAT (Figure S27a). As for ^{13}C NMR spectra for DAT substrate, only one carbon signal was detected at δ=158.3 ppm in the ^{13}C NMR spectrum for DAT, which can be assigned to carbon atoms in the triazole unit (Figure S27b).

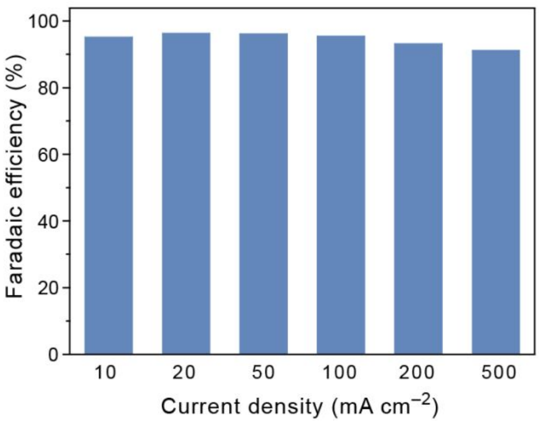

Figure S28. Faradaic efficiency of DAT-OCD at various current densities.

Table S1. EXAFS fitting parameters at the Pt L_3-edge for $Pt_1@VS_2$ and Pt foil reference

**Samples**	Shell	N [a]	R (Å) [b]	σ^2 ($Å^2 \cdot 10^{-3}$) [c]	ΔE^0 (eV) [d]	R factor (%)
$Pt_1@VS_2$	Pt−S	3.1 (±0.4)	2.51 (±0.01)	4.2	-3.7	0.8
	Pt−V	3.0 (±0.2)	3.20 (±0.01)	3.8	-4.5	
Pt foil (25 °C)	Pt−Pt (in metallic state Pt)	12	2.76	4.1	8.1	0.06

[a] N: coordination number; [b] R: bond distance; [c] σ^2: Debye-Waller factor; [d] ΔE_0: the inner potential correction.

R factor: goodness of fit. S_0^2, 0.779, was obtained from the experimental EXAFS fitting over Pt foil reference with known crystallographic value, which was then used to all the samples.

Table S2. Comparison of potential at 10 mA cm^{-2} for anodic oxidation reaction to upgrade chemicals with recently reported hybrid water electrolysis

Substrate	Electrode materials	Potential at 10 mA cm^{-2} (V vs. RHE)	Reference
**3,5-Diamino-1*H*-1,2,4-triazole (DAT)**	**CuO@CF NWs**	**0.87**	***This Work***
5-Hydroxymethylfurfural (HMF)	Cobalt hydroxide/Ru	1.0	*Angew. Chem. Int. Ed. 2024, e202319642*
Glycerol	Ru@MnO_{2-x}	1.13	*Angew. Chem. Int. Ed. 2023, e202314569*
HMF	Rh-SA/NiFe NMLDH	1.22	*J. Am. Chem. Soc. 2023, 145, 32, 17577–17587*
Glycerol	NiVRu-LDHs NAs/NF	1.24	*Adv. Mater. 2023, 35, 2300935*
Ethylene Glycol (EG)	$CoNi_{0.25}P$	1.25	*Nat. Commun. 2021, 12,4679*
Benzyl Alcohol (BAl)	NC@CuCo Nitride	1.25	*Adv. Funct. Mater. 2017, 27, 1704169*
Glycerol	HEA-CoNiCuMnMo	1.25	*J. Am. Chem. Soc. 2022, 144, 16, 7224–7235*
Sterol	Cr-Ni_3N/GF	1.25	*Angew. Chem. Int. Ed. 2023, e202306553*
Lignocellulose	Ir-NiFeO@NF	1.30	*Appl. Catal. B: Environ. 2023, 336, 122937*
HMF	FeSi	1.31	*Adv. Mater. 2021, 33, 2008823*
Tetrahydroisoquinolines (THIQs)	CoFe-$NiSe_2$	1.31	*Angew. Chem. Int. Ed. 2023, 62, e202216347*
EG	$CoSe_2$-Ni	1.31	*Adv. Mater. 2024, 2312618*
HMF	NiFe	1.32	*Angew. Chem. Int. Ed. 2022, e202215804*
Methanol	NiMnLDH	1.34	*Nat. Commun. 2023, 14:1686*
Benzylamine (BA)	Mn-$Ni(OH)_2$	1.34	*J. Am. Chem. Soc. 2022, 144, 15185-15192*
BA	NiSe	1.35	*Angew. Chem. 2018, 130, 13347–13350*
Bal	$Ni(OH)_2$	1.35	*Sci. Adv. 2024, 10, eadn0947*

Cycloalkanol	$Co_2(OH)_3Cl/FeOOH$	1.35	*Chem. Eng. J. 2022, 442, 136264*
Ethanol	NiOOH-CuO	1.35	*Appl. Catal. B: Environ. 2023, 325, 122388*
Methanol	$Mo\text{-}Co_4N$	1.356	*J. Mater. Chem. A, 2021, 9, 21094–21100*
BA	$Fe\text{-}Ni_3S_2$	1.36	*Adv. Funct. Mater. 2023, 2301884*
Glycerol	MnO_2/CP	1.36	*Angew. Chem. Int. Ed. 2021, 60, 21464–21472*
Pyrazole	Ni-MOF	1.37	*Angew. Chem. Int. Ed. 2024, e202402176*
BA	$CoSe_2$/Ni−SVs SBs	1.37	*ACS Catal. 2022, 12, 11391-11401*
Methanol	h-NiSe/CNTs	1.37	*Adv. Funct. Mater. 2021, 31, 2008812*
Cyclohexenylethylamine (CE)	NiO/NC	1.38	*Adv. Energy Mater. 2024, 2400374*
Glycerol	Ni-phen	1.38	*Angew. Chem. Int. Ed. 2023, e202216083*
Methanol	$CoPt_3@Co_2P/Co@NCNT$	1.43	*Small 2021, 2104656*
Cyclohexanone (CH)	$Ni(OH)_2$-SDS	1.45	*Nat. Commun. 2022, 13, 5009*
Ethanol	VR-β-$Ni(OH)_2$	1.45	*Angew. Chem. Int. Ed. 2023, e202316449*

References

(1) Kresse, G.; Furthmiiller, J. Efficiency of Ab-Initio Total Energy Calculations for Metals and Semiconductors Using a Plane-Wave Basis Set. *Comput. Mater. Sci.* **1996**, *6*, 15–50.

(2) Mathew, K.; Sundararaman, R.; Letchworth-Weaver, K.; Arias, T. A.; Hennig, R. G. Implicit Solvation Model for Density-Functional Study of Nanocrystal Surfaces and Reaction Pathways. *J. Chem. Phys.* **2014**, *140* (8), 084106.
